# TEAD1 Enhances Exosome Secretion and Promotes Exosome‐Mediated Tissue Regeneration

**DOI:** 10.1002/advs.202514104

**Published:** 2026-03-12

**Authors:** Yan Pu, Yi Wan, Wenhao Shi, Bin Li, Haiyue Zhang, Jun Wu, Lingling Li, Wenjia Guo, Chen Ding, Wenjun Yang

**Affiliations:** ^1^ Clinical Research Center for Cell‐based Immunotherapy of Shanghai Pudong Hospital Fudan University Pudong Medical Center State Key Laboratory of Genetics and Development of Complex Phenotypes School of Life Sciences Human Phenome Institute Fudan University Shanghai China; ^2^ Analysis Center Chemistry Department Tsinghua University Beijing China; ^3^ Department of Spine Surgery Xin Hua Hospital Affiliated to Shanghai Jiao Tong University School of Medicine Shanghai China; ^4^ Department of Cancer Research Institute Xinjiang Key Laboratory of Translational Biomedical Engineering Affiliated Cancer Hospital of Xinjiang Medical University Urumqi China; ^5^ CAS Center for Excellence in Molecular Cell Science Cell Bank Shanghai Institute of Biochemistry and Cell Biology Chinese Academy of Sciences University of Chinese Academy of Sciences Shanghai China; ^6^ Department of Pediatric Orthopedics Xin Hua Hospital Affiliated to Shanghai Jiao Tong University School of Medicine Shanghai China

**Keywords:** exosome, spinal cord injury repair, TEAD1, wound healing

## Abstract

Exosomes serve as intercellular communication vectors and are involved in a broad range of physiological functions. Although exosome‐based therapies have demonstrated diverse functional potential, the regulatory mechanisms underlying their biogenesis and secretion remain poorly understood. Here, we report that TEAD1 functions as a molecular switch, dramatically enhancing the synthesis and secretion of exosomes. Mechanistically, TEAD1 enhances exosome secretion by promoting the expression of exosome secretion‐associated proteins RAB11, CD9, and SNAP23. We found that TEAD1 enhances exosome secretion from adipose‐derived mesenchymal stem cells, thereby promoting skin wound healing in diabetic mice. Similarly, TEAD1 promotes the release of exosomes from bone marrow‐derived mesenchymal stem cells, thereby facilitating spinal cord injury (SCI) repair. Our study elucidates a novel role for TEAD1 in driving exosome secretion in different cell types, highlighting the therapeutic potential of TEAD1 in enhancing tissue regeneration, particularly in diabetic wound healing and SCI repair.

## Graphical abstract

TEAD1 functions as a crucial molecular switch regulating exosome secretion in various cell types. TEAD1 enhances exosome secretion by upregulating key proteins associated with exosome secretion, including RAB11, CD9, and SNAP23. This study reveals a novel role for TEAD1 in regulating exosome secretion and tissue regeneration, particularly in diabetic wound healing and spinal cord injury models.

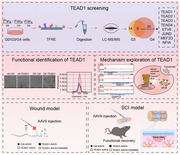

## 1 Introduction

Exosomes are nanosized extracellular vesicles secreted by various cell types. They are typically round in shape, ranging from ∼40 to 160 nm in diameter (average ∼100 nm), and are enclosed by a double‐layered phospholipid membrane containing diverse bioactive molecules such as lipids, proteins, and nucleic acids. Exosomes mediate critical biological signaling processes, influencing systemic physiology and facilitating intercellular communication. Moreover, they are involved in crucial physiological and pathological processes such as nervous system development, apoptosis regulation, inflammation, autophagy, and oxidative stress [1].

Due to their unique ability to modulate immune responses, transfer functional biomolecules, and facilitate tissue repair, exosomes have emerged as promising therapeutic agents in regenerative medicine [2]. Their role has been extensively investigated in a wide range of pathological conditions, including skin wounds, spinal cord injury (SCI), cardiovascular diseases, neurodegenerative disorders, and musculoskeletal damage. For example, mesenchymal stem cell‐derived exosomes have shown therapeutic potential in a broad range of conditions, including neurological disorders, cardiovascular disease, wound healing, and aesthetic dermatology. Clinical trials involving adipose‐derived stem cell exosomes (ADSC‐Exos) have demonstrated significant improvements in wrinkle reduction [3], skin hydration [4], and overall skin texture [5]. In dermatological applications, exosomes accelerate wound healing by promoting collagen synthesis, reducing inflammation, and enhancing neovascularization, particularly in chronic wounds such as diabetic ulcers [6, 7]. In neuroregeneration, exosomes have shown efficacy in mitigating neurodegenerative conditions such as Alzheimer's and Parkinson's disease by crossing the blood–brain barrier, reducing neuroinflammation, and promoting neuronal regeneration [8]. Given these multifaceted therapeutic effects, exosomes have garnered increasing attention as promising candidates for applications in tissue regeneration and regenerative medicine [9]. Despite the growing interest and encouraging preclinical data, several critical challenges continue to limit the clinical translation of exosome‐based therapies. The mechanisms underlying exosome biogenesis, cargo sorting, and cellular uptake remain incompletely understood. Moreover, large‐scale exosome production and difficulties in storage and transportation remain a significant bottleneck [10, 11]. Currently, most exosome production occurs under research‐grade conditions in academic or early‐stage biotech laboratories, with only a limited number of Contract Development and Manufacturing Organizations providing cGMP‐compliant production services [12, 13]. To improve exosome yields, various strategies have been explored, including hypoxia, 3D culture systems, and stress induction. While these methods can stimulate vesicle release, they often compromise the quality and consistency of the final product [14]. Genetic engineering approaches, combining Rab4 knockdown with supplementation using red cell membrane particles, have been shown to increase yield, though their clinical feasibility remains uncertain [15]. Chemical stimulation using calcium ionophores or small molecules can also enhance short‐term exosome release but may induce cytotoxicity and lead to undesirable alterations in exosome content [16]. High‐density bioreactor‐based culture systems offer a scalable alternative, yet maintaining exosome purity and functionality under these conditions remains challenging [17]. To date, no effective method addresses the need for high‐yield, cost‐efficient, and clinically compliant exosome production.

Exosome biogenesis and secretion are highly regulated processes involving multiple molecular machineries. Among the best‐characterized are the endosomal sorting complexes required for transport (ESCRT), which mediate the inward budding of the endosomal membrane to form intraluminal vesicles (ILVs), and SNARE proteins, which facilitate the fusion of multivesicular bodies with the plasma membrane to release exosomes. Other regulators, such as Rab GTPases and tetraspanins, also contribute to distinct steps of vesicle trafficking and cargo sorting [18].

Exosome biogenesis and secretion processes are tightly regulated by multiple cellular pathways. Transcription factors (TFs) play a pivotal role in modulating exosome production by regulating the expression of genes involved in endosomal sorting, MVB formation, and vesicle trafficking. For example, the tumor suppressor p53 has been shown to regulate exosome release under stress conditions by modulating the transcription of a subset of target genes [19]. However, the regulatory effect of p53 on exosome secretion appears to be indirect and is often accompanied by alterations in exosomal cargo composition, limiting its utility as a precise molecular switch. Similarly, STAT3 has been implicated in promoting exosome release through the phosphorylation of PKM2 and SNAP23, thereby facilitating SNARE complex formation [20]. Yet, this mechanism also reflects a signaling cascade that is not directly or specifically tailored to exosome biogenesis, and the magnitude of enhancement in secretion remains relatively modest. These findings reveal an incomplete understanding of transcription factor regulation of exosome biogenesis and highlight the need to identify more specific and effective upstream regulators. Understanding the transcriptional regulation of exosome biogenesis provides valuable insights into their roles in health and disease. It offers potential avenues for therapeutic intervention and biomarker discovery, emphasizing the importance of TFs in modulating exosome‐mediated intercellular communication.

In this study, we identified TEAD1, a transcription factor, as a novel regulator of exosome biogenesis and secretion. We demonstrated that TEAD1 functions as a molecular switch, markedly enhancing the synthesis and release of exosomes by upregulating the expression of key proteins involved in exosome secretion, including RAB11, CD9, and SNAP23. Our findings reveal a previously unrecognized role for TEAD1 in promoting exosome‐mediated tissue regeneration in multiple cell types. In particular, TEAD1 enhances exosome secretion from adipose‐derived stem cells (ADSCs) and promotes skin wound healing in diabetic mice. Additionally, TEAD1 enhances exosome secretion from bone marrow‐derived stem cells (BMSCs) and promotes the repair of SCI. We utilized adeno‐associated virus‐mediated TEAD1 (TEAD1‐AAV) to achieve localized in situ delivery of exosomes in an in vivo model, thereby promoting tissue repair. These results not only highlight the therapeutic potential of TEAD1 in regenerative medicine, particularly for treating chronic wounds and neural injuries, but also suggest that exosome‐based therapies regulated by TEAD1 could be extended to a broader range of diseases that require tissue regeneration and repair.

## 2 Results

### 2.1 TF Activity Profiles Reveal TEADs as Key TFs Regulating Exosome Secretion

**FIGURE 1:**
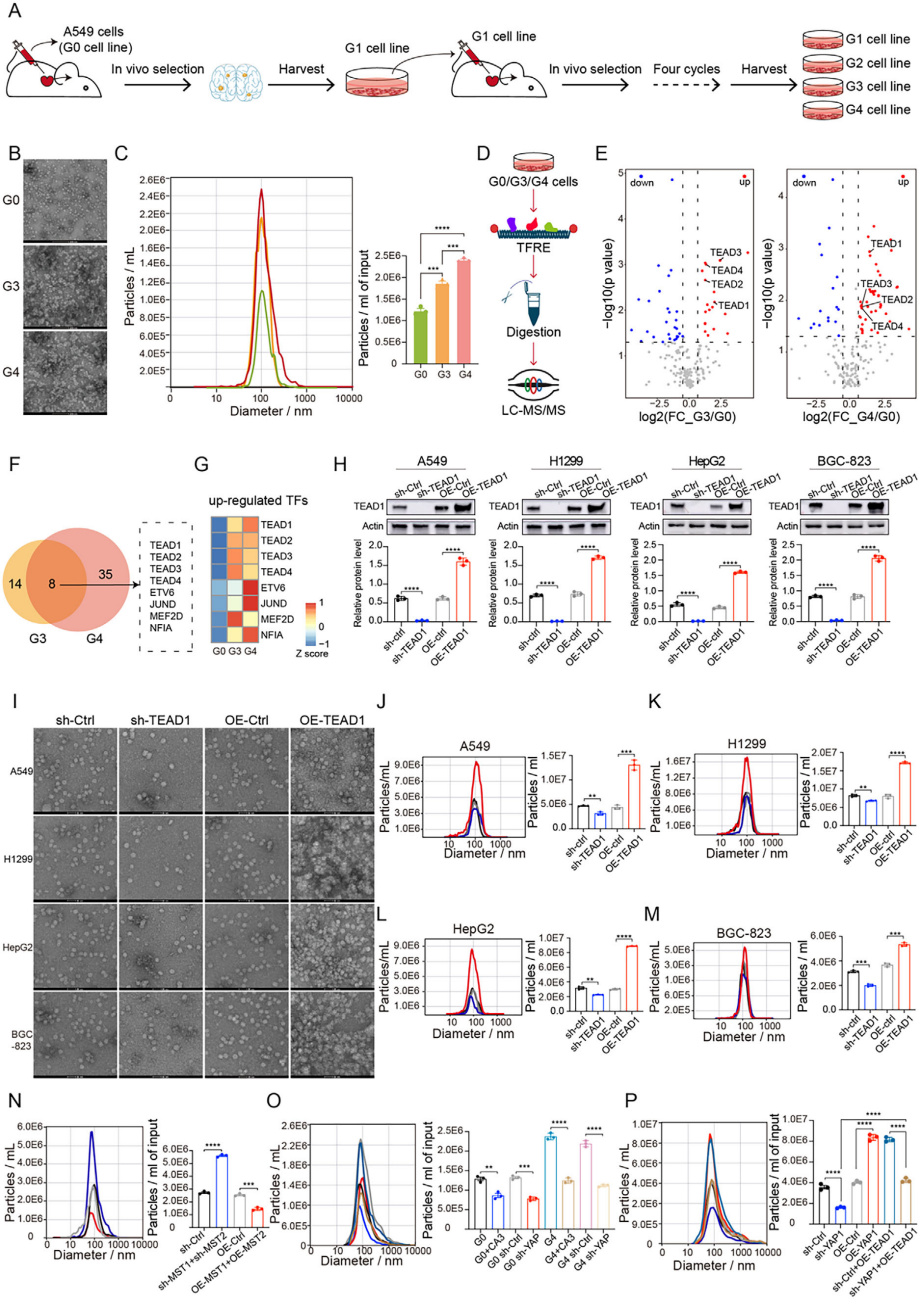
TEADs are key molecules regulating the synthesis and secretion of exosomes. (A) Construction of high‐ and low‐migratory lung cancer cell lines (G0, G3, and G4, respectively). G0 represents the low‐migratory cell line, while G4 represents the high‐migratory cell line. (B) TEM images of exosomes isolated from cell culture supernatants. Scale bar = 200 nm. (C) NTA of exosome size distribution and concentration (*n* = 3 per group, mean ± SD). (D) Workflow of transcription factor activity proteomics in lung cancer cell lines of different malignancies. (E) Comparison of transcription factor activities among lung cancer cell lines of different malignancies. The figure shows the comparison of transcription factor activities between G3 and G4 cells versus G0 cells. Red indicates up‐regulated TFs, while blue indicates down‐regulated ones. (F) Overlap of up‐regulated TFs in G3 and G4 cell lines. (G) Activity distribution of eight up‐regulated TFs in G3 and G4 cells. (H) Western blot validation of TEAD1 overexpression and Knockdown in four different tumor cell lines (*n* = 3 per group, mean ± SD). (I) TEM images of exosomes after TEAD1 knockdown and overexpression in four different cell lines. Scale bar = 50 nm. (J‐M) Statistics of exosome size and particle number after TEAD1 knockdown and overexpression in four different cell lines (*n* = 3 per group, mean ± SD). (N) Statistics of exosome size and particle number in H1299 cell lines after MST1/2 knockdown or overexpression (*n* = 3 per group, mean ± SD). (O) Statistics of exosome size and particle number in G0 and G4 cell lines after CA3 treatment or YAP knockdown (*n* = 3 per group, mean ± SD). (P) Statistics of exosome size and particle number after TEAD1 overexpression in sh‐YAP H1299 cell lines (*n* = 3 per group, mean ± SD). Differences among the groups were determined with one‐way ANOVA with Tukey's posttest and Student's two‐tailed t‐test. Data were considered statistically significant when *p* < 0.01 (∗∗), *p* < 0.001 (∗∗∗), and *p* < 0.0001 (∗∗∗∗) versus the indicated group.

In this study, we established an in vivo metastasis model using lung cancer cells to progressively select for enhanced metastatic capacity across successive cell generations. Specifically, A549 cells (designated as G0) were injected intracardially, leading to spontaneous metastases in the brain, bones, and other organs. Once brain metastases were established, within 4 to 8 weeks, metastatic lesions were surgically resected and cultured in vitro to derive the next‐generation cell line (G1). This selection process was iteratively repeated to generate G2, G3, and G4 cell lines, each representing progressively enriched metastatic potential (Figure 1A). Previous studies had shown that highly metastatic tumor cells often secreted increased quantities of exosomes that contributed to the formation of pre‐metastatic niches and promoted organotropic metastasis [21, 22]. We next investigated whether exosome secretion was altered across these progressively metastatic cell generations. Transmission electron microscopy (TEM) and nanoparticle tracking analysis (NTA) revealed significant differences in the number of exosomes released by the three distinct lung cancer cell lines, while their exosome sizes remained comparable. Notably, exosome secretion was markedly elevated in the G3 and G4 generations compared to the G0 cell line. The G3 cell line released exosomes approximately 1.5‐fold more than the G0 cell line, while the G4 cell line released exosomes approximately 2.1‐fold more than the G0 cell line (Figure 1B,C; Figure S1A). Tumor‐derived exosomes play a critical role in promoting metastasis through multiple mechanisms [18, 21]. To determine whether the exosomes secreted by the G4 cell line possessed migrating‐promoting functions, we performed transwell assays. After treatment of the G0 cell line with exosomes derived from either the G0 or G4 cell lines for 24 h, we found that exosomes derived from the G4 cell line displayed enhanced migratory capacities compared to exosomes derived from the G0 cell line (Figure S1B). These observations underscored that treating tumor cells with exosomes from the G4 cell line potentiates tumor metastasis, independent of the cell's intrinsic metastatic potential, suggesting that exosomes secreted by the G4 cell line exhibited metastatic functions. These findings demonstrate that the G4 cell line exhibits the highest level of exosome secretion among all tested generations.

TFs play pivotal roles in diverse biological processes as central regulatory components of signal transduction networks [23]. Emerging evidence indicates that TFs also regulate exosome biogenesis and secretion by modulating the expression of genes involved in vesicle trafficking and membrane dynamics [18, 19]. To systematically profile TF activity under varying conditions, we employed Transcription Factor Response Element (TFRE) profiling, a MS‐based proteomic approach that captures TFs by TF response elements DNA from nuclear extracts, enabling comprehensive characterization of TF activity landscapes in relation to exosome production [24]. Using this TFRE‐based proteomic approach, we assessed the TF activity profiles in lung cancer cells across different generations (G0, G3, and G4) (Figure 1D). By comparing the proteomic profiles of TF activity, we identified distinct alterations in TF regulatory landscapes in both G3 and G4 groups, each relative to G0 groups (Figure 1E; Table S1a). Specifically, we utilized the Wilcoxon rank‐sum test to identify differentially expressed proteins (DEPs), using a threshold of fold change > 2 and *p* < 0.05. As a result, 22 TFs were significantly upregulated in the G3 cell line, and 43 TFs were upregulated in the G4 cell line (Figure 1F). Among these, 8 TFs were commonly upregulated in both G3 and G4 groups, including four members of the TEAD family (TEAD1, TEAD2, TEAD3, and TEAD4). Notably, TEAD1 exhibited significant upregulation in the G4 cell line (Figure 1G; Figure S1C,D). These findings suggested that TEAD1 might play a pivotal role in promoting exosome biogenesis and secretion.

To investigate the functional role of TEAD1 in exosome secretion, we established TEAD1 overexpression (OE‐TEAD1) and knockdown (sh‐TEAD1) cell lines using lung cancer cell lines H1299 and A549 cells (Figure 1H; Figure S2A). Exosomes were then isolated from these cell lines for subsequent analyses (Figure S2B,C). As shown in Figure 1I–K, OE‐TEAD1 led to a marked increase in exosome secretion in both H1299 and A549 cells, whereas sh‐TEAD1 resulted in a significant reduction compared to the control group. These results suggested that TEAD1 played a crucial role in regulating exosome secretion in lung cancer cells. In addition to verifying the function of TEAD1 in lung cancer cell lines, we further investigated whether TEAD1 played a similar role in promoting exosome production in other tumor cell types. Specifically, we established TEAD1 overexpression and knockdown models in the hepatocellular carcinoma cell line HepG2 and the gastric cancer cell line BGC‐823, and confirmed the efficiency of overexpression or knockdown by assessing TEAD1 mRNA and protein levels (Figure 1H; Figure S2A). Consistent with our findings in lung cancer cell lines, OE‐TEAD1 significantly enhanced exosome secretion, whereas sh‐TEAD1 markedly reduced exosome secretion in HepG2 and BGC‐823 cells (Figure 1I,L,M). These results demonstrated that TEAD1 played a pivotal role in promoting exosome biogenesis and secretion in multiple types of cells.

Given the conserved pro‐exosomal role of TEAD1, we next examined whether other TEAD family members, including TEAD2, TEAD3, and TEAD4, exhibit similar regulatory effects. Specifically, we constructed overexpression (OE‐TEAD2, OE‐TEAD3, and OE‐TEAD4) and knockdown (sh‐TEAD2, sh‐TEAD3, and sh‐TEAD4) models in A549 lung cancer cells (Figure S2D,E). TEM revealed that all isolated exosomes displayed the typical cup‐shaped morphology with diameters of 70–150 nm, indicating that modulation of these TFs did not alter vesicle structure (Figure S2F). NTA revealed that overexpression of TEAD2, TEAD3, and TEAD4 all promoted exosome secretion, whereas knockdown inhibited exosome secretion. Notably, TEAD1 overexpression led to a pronounced increase, with exosome levels at least 3.2‐, 2.4‐, and 2.7‐fold higher than those observed in the TEAD2, TEAD3, and TEAD4 overexpression groups, respectively (Figure S2G). These data identify TEAD1 as the dominant TEAD family member driving exosome biogenesis. To further investigate whether other TFs, such as members of the STAT family (STAT1, STAT3, and STAT6), MYC, MEF2D, JUND, NFκB, ETV6, NFIA, and P65, might also contribute to enhanced exosome secretion, thereby assessing the selective role of TEAD1, we performed comparative activity profiling based on TFRE proteomic data (Figure S1C). Boxplot analyses were conducted for four TEAD family members (TEAD1, TEAD2, TEAD3, and TEAD4), three STAT family members (STAT1, STAT3, and STAT6), as well as MYC, JUND, NFκB, and P65 across G0, G3, and G4 lung cancer cells. As shown in Figure S1D, TEAD1 exhibited the highest activity in G4 cells, consistent with its potential role in driving exosome secretion. Additionally, MEF2D, JUND, ETV6, and NFIA displayed moderate increases in activity in G3 and G4 cells. In contrast, STAT family members (STAT1, STAT3, and STAT6), NFκB, and c‐MYC showed no significant changes in activity among the three generations, suggesting that their transcriptional outputs are unlikely to be associated with the enhanced exosome secretion phenotype. Despite the lack of significant variation in these classical TFs, we remained intrigued by whether these candidates might exert subtle or context‐dependent influences on exosome biogenesis. Next, we investigated whether these TFs that were well‐characterized oncogenic TFs, such as JUND, MYC, P65, STAT1, STAT3, and STAT6, might play functional roles in exosome secretion. We established corresponding overexpression (OE‐JUND, OE‐MYC, OE‐P65, OE‐STAT1, OE‐STAT3, and OE‐STAT6) and knockdown (sh‐JUND, sh‐MYC, sh‐P65, sh‐STAT1, sh‐STAT3, and sh‐STAT6) models in A549 cells (Figure S3A,B). Exosomes were isolated from the above cell lines and characterized by TEM and NTA. TEM analysis confirmed that vesicle morphology remained unaltered upon manipulation of these TFs (Figure S3C). NTA revealed that STAT1 overexpression modestly enhanced exosome secretion, while inhibiting STAT1 expression could block exosome secretion, with the overall changes remaining relatively modest (Figure S3D). In contrast, JUND, MYC, P65, STAT3, and STAT6 overexpression did not induce any detectable change in exosome production (Figure S2G). Although STAT1 overexpression resulted in a modest increase in exosome release, no alterations in STAT‐related TFRE signals were observed in A549 cell lines with different generations (G0, G3, and G4), suggesting an indirect regulatory mechanism (Figure S1D). The underlying mechanisms responsible for this discrepancy warrant further investigation in future studies.

YAP/TAZ are nuclear effectors of the Hippo pathway. YAP, a transcriptional co‐activator, regulates gene expression primarily through binding to TEAD family TFs, including TEAD1 [25]. When the Hippo pathway is inactivated, dephosphorylated YAP/TAZ translocates into the nucleus and binds to the TFs TEAD1‐4 to induce gene expression [26]. To elucidate how upstream regulators and downstream effectors of the Hippo pathway contribute to the elevated TEAD1 levels observed in G0 and G4 cells, we performed proteomic profiling in both cell lines. We found that the protein levels of the core components MST1 (also known as STK4), MST2 (also known as STK3), LATS1, and LATS2 in the Hippo signaling pathway were significantly reduced in G4 cells compared to G0 cells. In contrast, the protein levels of the downstream effectors YAP and TEAD1 in the Hippo signaling pathway were significantly elevated in G4 cells compared to G0 cells, indicating the inactivation of the Hippo pathway core components MST1/2 and LATS1/2 in G4 cells (Figure S3A). To further validate the proteomic profiling results, we performed Western blot analyses to analyze the expression levels of core Hippo pathway components in G0 and G4 cells. Consistent with the proteomic data, the protein levels of the upstream regulators MST1, MST2, LATS1, and LATS2 were significantly reduced in G4 cells compared with G0 cells. The downstream effectors YAP/TAZ and TEAD1 were substantially upregulated in G4 cells, while the expression level of YAP‐S127 was markedly reduced (Figure S3B). These results further suggest that in G4 cells, inactivation of the upstream Hippo regulators MST1/2 and LATS1/2 promotes YAP dephosphorylation, which likely facilitates its nuclear translocation, enabling YAP to bind TEAD1 and enhance TEAD1 expression compared to G0 cells.

To further investigate the impact of upstream Hippo regulators MST1 and MST2 on the downstream effectors and exosome secretion, we established MST1/2 knockdown and overexpression cell lines in H1299 cells. First, we examined the effects of MST1/2 perturbation on the protein expression of downstream Hippo components and TEAD1 by Western blotting. We found that knockdown of MST1 and MST2 resulted in a marked decrease in LATS1, LATS2, and phosphorylated YAP (YAP‐S127), whereas YAP, TAZ, and TEAD1 levels were significantly increased. Conversely, overexpression of MST1 and MST2 led to increased levels of LATS1, LATS2, and YAP‐S127, with concomitant reduction in YAP, TAZ, and TEAD1 expression (Figure S3C). Next, to assess the functional consequences of MST1/2 modulation on exosome secretion, we isolated exosomes from cell culture supernatants and analyzed them using TEM and NTA. TEM analysis showed no discernible changes in exosome morphology following MST1/2 knockdown or overexpression (Figure S3D). In contrast, NTA revealed that MST1/2 knockdown significantly enhanced exosome secretion, whereas MST1/2 overexpression markedly suppressed exosome release (Figure 1N).

To elucidate the mediating roles of YAP and TAZ in TEAD1 transcriptional output, we investigated exosome secretion following YAP/TAZ inhibition or YAP knockdown in G0 and G4 cells. First, G0 and G4 cells were treated with the YAP/TAZ inhibitor CA3 [27]. Western blot showed that CA3 treatment markedly reduced YAP and TEAD1 protein levels in both G0 and G4 cells relative to the control groups (Figure S5A). Exosomes were subsequently isolated from these cell lines and characterized by TEM and NTA. TEM analysis revealed that CA3 treatment did not affect the morphology or size distribution of exosomes in either G0 or G4 cells (Figure S5B). In contrast, NTA revealed that CA3 treatment markedly reduced the total number of secreted exosomes compared with the control group (Figure 1O). Next, to directly assess the contribution of YAP and TAZ to TEAD1‐mediated transcriptional regulation, YAP was knocked down in the G0 and G4 cells, with the efficiency of YAP knockdown confirmed by Western blotting (Figure S5A), followed by analysis of exosome secretion. Consistent with the pharmacological inhibition results, genetic silencing of YAP in both G0 and G4 cells resulted in a pronounced decrease in exosome release, without affecting particle size or structural integrity (Figure 1O; Figure S5B). These findings demonstrate that YAP plays a critical role in mediating TEAD1‐driven exosome biogenesis, and inhibition of the YAP/TAZ pathway, either pharmacologically or genetically, significantly suppresses exosome secretion in lung cancer cells.

Given the potential clinical relevance of these findings, we further tested IAG933, a clinically relevant YAP/TAZ–TEAD inhibitor currently in Phase I trials, to determine whether pharmacological disruption of this pathway similarly suppresses exosome secretion. To elucidate the mediating roles of YAP and TAZ in TEAD1 transcriptional output, we investigated exosome secretion following YAP/TAZ inhibition in lung cancer cell lines with different metastatic capacities, G0 and G4. First, G0 and G4 cells were treated with IAG933 (Target Mol, T77725), a commercially available YAP/TAZ‐TEAD inhibitor currently in Phase I clinical trials. Western blot showed that IAG933 treatment moderately reduced YAP and TEAD1 protein levels in both G0 and G4 cells relative to the control groups (Figure S5C). Exosomes were subsequently isolated from these cell lines and characterized by TEM and NTA. TEM analysis revealed that IAG933 treatment did not affect the morphology or size distribution of exosomes in either G0 or G4 cells (Figure S5D). In contrast, NTA revealed that IAG933 treatment significantly reduced the total number of secreted exosomes compared with the control group (Figure S5E). These findings demonstrate that YAP plays a critical role in mediating TEAD1‐driven exosome biogenesis, and inhibition of the YAP/TAZ‐TEAD1 pathway significantly suppresses exosome secretion in lung cancer cells.

To further elucidate the mediating roles of YAP and TAZ in TEAD1 transcriptional output, we constructed both YAP knockdown (sh‐YAP) and overexpression (OE‐YAP) H1299 cell lines and the corresponding controls (OE‐Ctrl and sh‐Ctrl, respectively), and confirmed the efficiency of overexpression or knockdown by assessing YAP mRNA and protein levels (Figure S5F,G). We found that TEAD1 expression was decreased in sh‐YAP and upregulated in OE‐YAP cells compared with controls (Figure S5G). We next examined whether YAP influences exosome secretion through TEAD1. Exosomes were isolated from the above cell lines and characterized by TEM and NTA. TEM analysis revealed that knockdown or overexpression of YAP did not affect exosome morphology or size distribution in H1299 cells (Figure S5H). In contrast, NTA demonstrated that, consistent with TEAD1 expression, OE‐YAP H1299 cells showed increased secreted exosomes, whereas sh‐YAP cells exhibited decreased exosome secretion compared with controls (Figure 1P). We then overexpressed TEAD1 in sh‐YAP cells and found that reintroducing TEAD1 rescued the reduction in exosome secretion caused by YAP knockdown (Figure 1P). These results indicate that YAP regulates exosome secretion in H1299 cells by modulating TEAD1 transcriptional activity.

To assess whether TEAD1 affected exosomal protein content, we performed proteomic analysis of exosomes isolated from control, OE‐TEAD1 H1299, and OE‐TEAD1 A549 cells. Among 1177 identified exosomal proteins, only 41 (32 upregulated, 9 downregulated; approximately 3.5% of the total proteins) showed differential expression (Wilcoxon rank‐sum test, *p* < 0.05, FC > 2; Figure S5I). Notably, exosomes derived from the OE‐TEAD1 group exhibited increased levels of key proteins associated with exosome biogenesis and secretion, such as RAB11, CD9, and SNAP23, consistent with the enhanced production of exosomes; the overall molecular composition of exosomes remained essentially unchanged, suggesting that TEAD1 did not affect overall cellular function (Figure S5J,K). These results demonstrated that TEAD1 promoted exosome production by upregulating key genes involved in exosome trafficking and release, specifically RAB11, CD9, and SNAP23, without significantly altering the composition of exosomal cargo.

### 2.2 TEAD1 Enhances Exosome Secretion by Directly Binding to the Promoters of RAB11, CD9, and SNAP23

**FIGURE 2:**
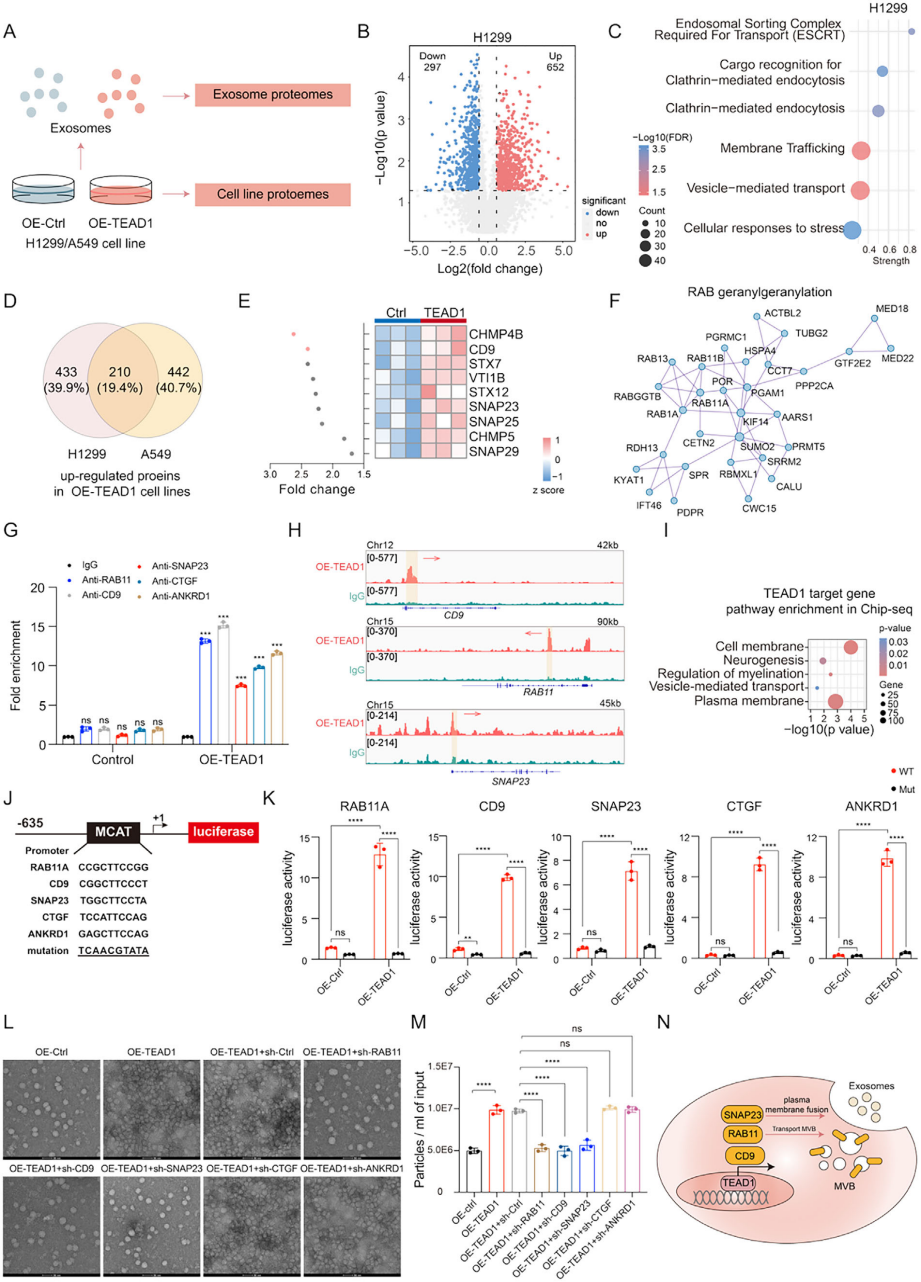
Proteomic analysis of changes in lung cancer cell lines and exosome‐related effects upon TEAD1 overexpression. (A) Proteomic study framework based on H1299 and A549 cell lines. (B) Protein abundance changes in H1299 cell lines upon TEAD1 overexpression. Red indicates up‐regulated TFs, while blue indicates down‐regulated ones. (C) Up‐regulated signaling pathways in H1299 cells overexpressing TEAD1. (D) Overlap of up‐regulated proteins in H1299 and A549 cell lines overexpressing TEAD1. (E) Fold change in abundance of nine proteins regulating exosome synthesis and secretion upon TEAD1 over‐expression (*n* = 3 per group). (F) Protein‐protein interaction network associated with RAB geranylgeranylation. (G,H) Chromatin immunosuppression (ChIP)‐quantitative polymerase chain reaction (G) and ChIP sequencing (H) illustrating the binding of TEAD1 to the promoter of RAB11, CD9, SNAP23, CTGF, and ANKRD1 (*n* = 3 per group, mean ± SD). (I) Pathway enrichment analysis of TEAD1 target genes. (J) Schematic diagram to show the generation of WT (wild type) or muscle CAT element (MCAT) mutation human RAB11, CD9, SNAP23, CTGF, and ANKRD1 promoter‐driven luciferase reporters. (K) Luciferase reporter assay of OE‐TEAD1 H1299 cells transfected with RAB11, CD9, SNAP23, CTGF, and ANKRD1 overexpression vector or empty vector control (*n* = 3 per group, mean ± SD). (L) TEM images of exosomes after RAB11, CD9, SNAP23, CTGF, and ANKRD1 knockdown in OE‐TEAD1 H1299 cell lines. Scale bar = 50 nm. (M) Statistics of exosome size and particle number after RAB11, CD9, SNAP23, CTGF, and ANKRD1 knockdown in OE‐TEAD1 H1299 cell lines. (*n* = 3 per group, mean ± SD). (N) Mechanistic model of TEAD1 promoting exosome synthesis and secretion. Differences among the groups were determined with one‐way ANOVA with Tukey's posttest and Student's two‐tailed t‐test. Data were considered statistically significant when *p* < 0.01 (∗∗), *p* < 0.001 (∗∗∗), and *p* < 0.0001 (∗∗∗∗) versus the indicated group.

To investigate the molecular mechanism by which TEAD1 promotes exosome secretion in lung cancer cell lines, we performed proteomic analyses on control, OE‐TEAD1 H1299, and OE‐TEAD1 A549 cells (Figure 2A). Proteomic profiling identified DEPs between the control and OE‐TEAD1 groups in both H1299 and A549 cells. Compared to the control group, in the H1299 cell, 652 proteins were upregulated in the OE‐TEAD1 group. In the A549 cell, 653 proteins were upregulated in the OE‐TEAD1 group (Wilcoxon rank‐sum test, *p* < 0.05, FC > 2, Figure 2B; Figure S6A and Table S2a). Pathway enrichment analysis revealed that, in the H1299 cell, the upregulated proteins in the OE‐TEAD1 group were significantly enriched in the ESCRT and clathrin‐mediated endocytosis pathways (Figure 2C). In the A549 cell, upregulated proteins in the OE‐TEAD1 group were predominantly enriched in the trans‐golgi network vesicle budding and golgi‐associated vesicle biogenesis pathways (Figure S6B,C). Notably, both cell lines exhibited significant enrichment of upregulated proteins in the cargo recognition for clathrin‐mediated endocytosis, membrane trafficking, and vesicle‐mediated transport pathways (Figure 2C; Figure S6B). These results suggested that OE‐TEAD1 significantly enhanced biological processes related to exosome synthesis, transport, and secretion in lung cancer H1299 and A549 cells. We further analyzed 210 proteins that were upregulated in both OE‐TEAD1 H1299 and A549 cells, and based on existing literature, upregulated proteins involved in exosome synthesis and secretion, including CHMP4B, CD9, STX7, VTI1B, STX12, and CHMP5, as well as members of the SNAP family (SNAP23, SNAP25, and SNAP29) and the RAB family (RAB1A, RAB3GAP2, RAB13, RAB9A, RAB9B, RAB27A, RABGGTB, RAB11A, and RAB11B) (Figure 2D–F; Figure S6D). Among these, the upregulation of RAB11, CD9, and SNAP23 was particularly significant. RAB11 has been reported to play a vital role in regulating MVB transport and influencing the docking process, while CD9 and SNAP23 contribute to the release of ILVs as exosomes. These results further indicated that TEAD1 enhanced exosome secretion by upregulating critical regulators, including RAB11, CD9, and SNAP23.

Based on these findings, we hypothesized that TEAD1 enhanced exosome secretion by transcriptionally activating key regulators involved in vesicle transport and membrane fusion, including RAB11, CD9, and SNAP23. To test this hypothesis, we first performed ChIP‐seq analysis, which revealed that TEAD1 was highly enriched at the promoter regions of these genes, exhibiting significantly stronger binding signals compared to the control group (Figure 2G,H; Figure S6E). Furthermore, YAP knockdown significantly decreased TEAD1 occupancy at the promoters of CD9, SNAP23, RAB11, CTGF, and ANKRD1, leading to reduced transcription of these genes. In contrast, overexpression of TEAD1 in sh‐YAP cells restored TEAD1 binding and the mRNA expression of CD9, SNAP23, RAB11, CTGF, and ANKRD1 (Figure S6F). These results suggested that TEAD1 might directly regulate their transcriptional activity. To further elucidate the functional relevance of these interactions, we conducted pathway enrichment analysis of TEAD1 target genes. The study revealed significant enrichment in pathways related to the plasma membrane and vesicle‐mediated transport, both of which were intimately linked to exosome biogenesis and secretion (Figure 2I).

TEAD1 exerts its transcriptional functions through recognizing conserved MCAT/TEAD binding motifs (typically 5′‐CATTCC‐3′ or related variants) within target‐gene promoters [28, 29]. These canonical TEAD/MCAT motifs represent the core DNA elements required for TEAD1 recruitment and YAP‐dependent transcriptional activation. To gain mechanistic insights, we next examined whether TEAD1 regulates exosome‐related genes through direct promoter binding. Bioinformatic motif scanning revealed that the promoter regions of RAB11, CD9, SNAP23, CTGF, and ANKRD1 all contain evolutionarily conserved TEAD/MCAT elements (Figure 2J), highly similar to those validated as functional TEAD1‐binding sites in published work [30]. To further determine whether TEAD1 activates these promoters in an MCAT‐dependent manner, we generated luciferase reporters containing the wild‐type promoter fragments of CD9, SNAP23, RAB11, CTGF, and ANKRD1 and corresponding constructs in which the TEAD/MCAT motifs were mutated (Figure 2J). These assays demonstrated that TEAD1 markedly enhanced the transcriptional activities of RAB11, CD9, and SNAP23, confirming its ability to upregulate their expression at the transcriptional level (Figure 2K). Furthermore, YAP overexpression significantly enhanced TEAD1‐dependent transcriptional activities, whereas YAP knockdown markedly suppressed transcriptional activation (Figure S6G). Notably, mutation of the MCAT elements substantially reduced the basal transcriptional activity and largely abolished TEAD1‐driven transcriptional activation (Figure 2K; Figure S6G).

To examine how YAP/TAZ activity influences TEAD1 expression and the transcriptional output of its downstream targets, we combined pharmacological inhibition and genetic silencing using the YAP/TAZ inhibitor CA3 and shRNA‐mediated YAP knockdown. We performed proteomic profiling in four groups of lung cancer cells: G0, G4, G0+CA3, and G4+CA3. DEPs were identified by comparing CA3‐treated cells with their untreated counterparts. We focused on well‐established TEAD1 targets(CTGF and ANKRD1 [31, 32]) and exosome‐related proteins revealed in our dataset (CD9, SNAP23, and RAB11). As shown in Figure S6H, CA3 treatment markedly reduced YAP and TEAD1 protein levels, accompanied by significant downregulation of both canonical TEAD1 targets and exosome‐associated proteins. To directly evaluate YAP/TAZ involvement, we performed YAP knockdown in G0 and G4 cells, generating G0, G4, G0+sh‐YAP, and G4+sh‐YAP groups. Proteomic analysis revealed that YAP depletion substantially decreased TEAD1 protein levels and reduced the expression of CTGF, ANKRD1, and exosome‐related proteins (RAB11, CD9, and SNAP23), consistent with the CA3‐induced proteomic changes (Figure S6I). We further validated these findings by Western blotting (Figure S7A). Both CA3 treatment and YAP knockdown led to pronounced reductions in TEAD1 protein abundance and concordant downregulation of its downstream targets, including classical TEAD1 genes and regulators of exosome secretion. These results demonstrate that YAP and TAZ play a critical mediating role in TEAD1 transcriptional activity. Inhibition of the YAP/TAZ signaling pathway, either pharmacologically or via genetic knockdown, significantly attenuates TEAD1 expression and its downstream transcriptional level, including those involved in exosome biogenesis. To further substantiate the role of YAP/TAZ in mediating TEAD1‐dependent transcriptional regulation, we employed an additional pharmacological inhibitor, IAG933. Western blot analysis showed that IAG933 treatment led to significant downregulation of canonical TEAD1 target genes (CTGF and ANKRD1) as well as exosome‐related proteins (RAB11, CD9, and SNAP23) in both G0 and G4 cells (Figure S7B). These findings demonstrate that YAP/TAZ activity is essential for sustaining TEAD1 expression and transcriptional output, thereby influencing the regulation of exosome biogenesis‐related pathways in lung cancer cells.

To determine whether these downstream targets functionally contribute to TEAD1‐mediated exosome secretion, we individually knocked down RAB11, CD9, and SNAP23 in OE‐TEAD1 H1299 cells. Exosomes were subsequently isolated for quantitative and morphological analyses. TEM and NTA confirmed that TEAD1 overexpression substantially increased exosome secretion relative to control cells. Notably, RAB11 knockdown effectively abolished the TEAD1‐induced enhancement of exosome secretion, while the size distribution of exosomes remained unchanged. Similarly, knockdown of CD9 or SNAP23 almost completely reversed the elevated exosome release observed in the OE‐TEAD1 group (Figure 2L,M; Figure S7C). To exclude the possibility that the observed effects were caused by canonical TEAD1 downstream genes, we also knocked down several well‐established TEAD1 downstream genes, including CTGF and ANKRD1, in OE‐TEAD1 H1299 cells. Interestingly, knockdown of these canonical TEAD1 targets did not affect exosome secretion, as assessed by both TEM and NTA analyses (Figure 2L,M; Figure S7C).

Taken together, these results provide strong evidence that TEAD1 promotes exosome secretion primarily through transcriptional activation of RAB11, CD9, and SNAP23, rather than via its classical downstream effectors (CTGF, ANKRD1). These findings identify RAB11, CD9, and SNAP23 as critical functional mediators of TEAD1‐driven exosome biogenesis and secretion (Figure 2N).

### 2.3 TEAD1‐Mediated Enhancement of Exosome Secretion Promotes Proliferation of HaCaT Cells and Angiogenesis of HUVECs

**FIGURE 3:**
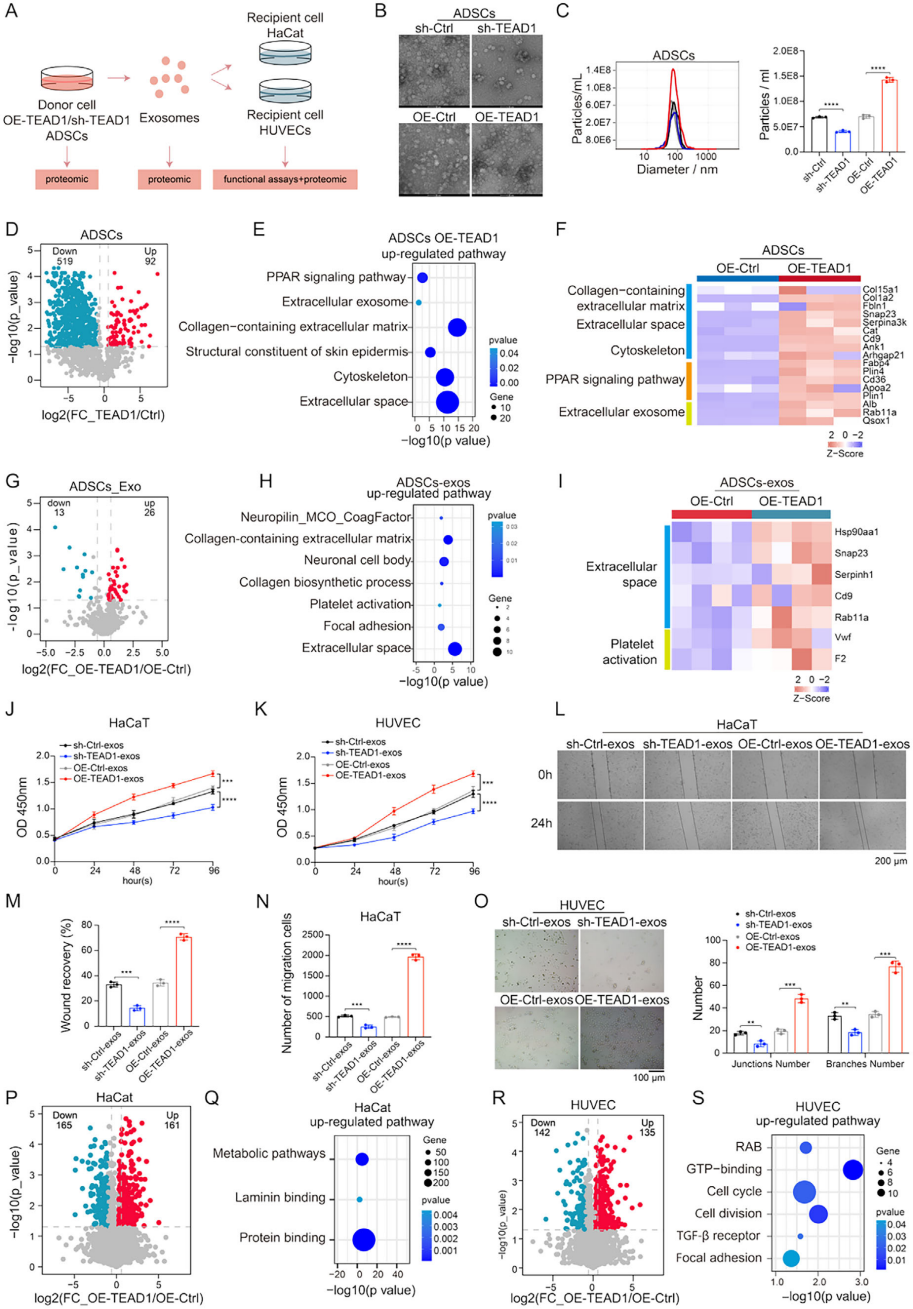
TEAD1‐mediated enhancement of exosome secretion promotes proliferation of HaCaT cells and angiogenesis of HUVECs. (A) Experimental design for assessing exosome‐mediated effects of TEAD1 modulation in ADSCs. (B) TEM images of exosomes after TEAD1 knockdown and overexpression in ADSCs. Scale bar = 50 nm. (C) Statistics of exosome size and particle number after TEAD1 knockdown and overexpression in ADSCs (*n* = 3 per group, mean ± SD). (D) Differential protein expression in ADSCs upon TEAD1 overexpression. Red indicates up‐regulated proteins, while blue indicates down‐regulated ones. (E) Up‐regulated signaling pathways in ADSCs overexpressing TEAD1. (F) The heatmap showing the DEPs in the OE‐TEAD1 and the OE‐Ctrl groups, the expression of proteins participating in the collagen‐containing extracellular matrix, extracellular space, cytoskeleton, PPAR signaling pathway, and extracellular exosome in OE‐TEAD1 and OE‐Ctrl cells is shown (*n* = 3 per group). (G) Differential protein expression in exosomes derived from ADSCs between the OE‐TEAD1 and the OE‐Ctrl groups. Red indicates up‐regulated proteins, while blue indicates down‐regulated ones. (H) Up‐regulated signaling pathways in exosomes derived from OE‐TEAD1 ADSCs. (I) The heatmap showing the DEPs in exosomes derived from ADSCs between the OE‐TEAD1 and the OE‐Ctrl groups, the expression of proteins participating in the extracellular space and platelet activation in OE‐TEAD1 and OE‐Ctrl cells is shown (*n* = 4 per group). (J,K) The proliferation assay results of HaCat cells (J) and HUVECs (K) treated with exosomes from different sources (*n* = 3 per group, mean ± SD). (L,M) The scratch assay results of HaCat cells treated with exosomes from different sources, scale bar, 200 µm (*n* = 3 per group, mean ± SD). (N) Transwell assays demonstrated the effects of exosomes from different sources on the migration of HaCat cells (*n* = 3 per group, mean ± SD). (O) A tube formation assay was performed to visualize the cell capillary network formation of HUVECs, scale bar, 100 µm (*n* = 3 per group, mean ± SD). (P) Differential protein expression in recipient cells (HaCat) treated with exosomes from OE‐TEAD1 ADSCs and OE‐Ctrl ADSCs. Red indicates up‐regulated proteins, while blue indicates down‐regulated ones. (Q) Up‐regulated signaling pathways in recipient cells (HaCat) treated with exosomes derived from OE‐TEAD1 ADSCs. (R) Differential protein expression in recipient cells (HUVECs) treated with exosomes from OE‐TEAD1 ADSCs and OE‐Ctrl ADSCs. Red indicates up‐regulated proteins, while blue indicates down‐regulated ones. (S) Up‐regulated signaling pathways in recipient cells (HUVECs) treated with exosomes derived from OE‐TEAD1 ADSCs. Differences among the groups were determined with one‐way ANOVA with Tukey's posttest and Student's two‐tailed t‐test. Data were considered statistically significant when *p* < 0.01 (∗∗), *p* < 0.001 (∗∗∗), and *p* < 0.0001 (∗∗∗∗) versus the indicated group.

MSCs‐derived exosomes play a pivotal role in tissue repair and regenerative medicine by facilitating intercellular communication and promoting cellular regeneration and healing processes [33]. To investigate whether TEAD1‐mediated enhancement of exosome production contributed to the reparative function, primary mouse ADSCs were isolated and subjected to Oil Red O staining to evaluate their adipogenic differentiation capacity (Figure S8A). We established OE‐TEAD1 and sh‐TEAD1 ADSC lines, and then isolated and identified their exosomes for further analysis (Figure 3A,B; Figure S8B–D). Quantitative analysis showed that OE‐TEAD1 markedly enhanced ADSC‐Exos secretion, whereas sh‐TEAD1 significantly reduced exosome secretion compared to the control group (Figure 3C).

To elucidate the regulatory role of TEAD1 in exosome secretion from ADSCs and its potential impact on tissue repair, we performed proteomic analyses on control and OE‐TEAD1 ADSC lines. The proteomic profiling identified DEPs between the control and the OE‐TEAD1 groups in ADSC lines (Figure 3D; Table S3a). Compared with the control group, the proteins upregulated in the OE‐TEAD1 group were mainly enriched in the PPAR signaling pathway, extracellular exosomes, collagen‐containing extracellular matrices, structural constituents of the skin epidermis, the cytoskeleton, and the extracellular space (Figure 3E,F; Table S3b). These findings suggested that TEAD1 enhanced ADSC‐derived exosome secretion and might promote tissue repair by upregulating proteins involved in extracellular matrix organization, cytoskeletal remodeling, and epidermal structure formation.

To assess whether TEAD1 affected the protein composition of exosomes, we performed proteomic profiling of exosomes isolated from control and OE‐TEAD1 ADSC lines. Among 583 identified exosomal proteins, only 39 (26 upregulated, 13 downregulated; approximately 6% of the total protein) showed differential expression (Wilcoxon rank‐sum test, *p* < 0.05, FC > 2; Figure 3G; Table S3c,d). Notably, the expression of key proteins involved in exosome biogenesis and secretion, such as RAB11, CD9, and SNAP23, was significantly increased in the OE‐TEAD1 group, consistent with the observed enhancement in exosome production. In contrast, the overall exosomal protein remained unchanged, indicating that TEAD1 selectively enhanced exosome production without broadly altering exosomal protein content or perturbing overall cellular function (Figure 3H,I).

To assess the biological effects of TEAD1‐regulated ADSC‐Exos on cell proliferation, wound healing, and angiogenesis, we conducted a series of in vitro experiments. Specifically, we evaluated whether exosomes derived from OE‐TEAD1 or sh‐TEAD1 ADSCs affected the viability, wound healing, and migration of HaCaT cells (human umbilical vein endothelial cells), as well as the viability and tube formation capacity of HUVECs (human keratinocytes) (Figure S8E,F). Cell viability measured by the Cell Counting Kit‐8 (CCK‐8) assay showed that OE‐TEAD1 ADSC‐Exos significantly enhanced the proliferation of both HaCaT cells and HUVECs compared to control ADSC‐Exos. In contrast, sh‐TEAD1 ADSC‐Exos did not exhibit such proliferative effects in both HaCaT cells and HUVECs compared to control ADSC‐Exos (Figure 3J,K). Consistently, wound healing and migration assays showed that OE‐TEAD1 ADSC‐Exos promoted wound closure and cell migration in HaCaT cells compared to control ADSC‐Exos, while sh‐TEAD1 ADSC‐Exos did not enhance these processes (Figure 3L–N). Similarly, in angiogenesis assays, OE‐TEAD1 ADSC‐Exos significantly enhanced tube formation in HUVECs, while sh‐TEAD1 ADSC‐Exos did not exert a promotive effect compared to control ADSC‐Exos (Figure 3O). Together, these findings highlighted TEAD1 as a key regulator of the regenerative functions of ADSC‐Exos.

To gain further insight into how OE‐TEAD1 ADSC‐Exos enhanced cell proliferation and angiogenesis, we conducted proteomic analyses independently on recipient HaCaT cells and HUVECs following co‐culture with either control or OE‐TEAD1 ADSC‐Exos. The proteomic profiling identified DEPs between HaCaT cells treated with control ADSC‐Exos and those treated with OE‐TEAD1 ADSC‐Exos (Figure 3P; Table S3e,f). Pathway enrichment analysis revealed that, compared to the control group, upregulated proteins in HaCaT cells treated with OE‐TEAD1 ADSC‐Exos were primarily involved in cell proliferation, laminin binding, and cell adhesion pathways (Figure 3Q; Figure S8G). Similarly, in HUVECs, DEPs were identified between cells treated with control ADSC‐Exos and those treated with OE‐TEAD1 ADSC‐Exos (Figure 3R; Table S3g,h). Pathway enrichment analysis showed that, compared to the control group, the upregulated proteins in HUVECs treated with OE‐TEAD1 ADSC‐Exos were mainly associated with cell cycle regulation, cell division, and cell adhesion pathways (Figure 3S; Figure S8H).

These findings suggested that TEAD1 enhanced the regenerative function of ADSC‐Exos by promoting their secretion and modulating their biological activity, thereby facilitating cellular processes essential for wound healing and angiogenesis.

### 2.4 Adeno‐Associated Virus‐Mediated TEAD1 (TEAD1‐AAV) Gene Therapy Enhances Skin Wound Healing by Promoting Exosome Secretion

We have demonstrated that TEAD1 significantly enhanced the secretion of exosomes from ADSCs, which in turn promoted endothelial cell proliferation and angiogenesis. These findings suggested that TEAD1‐mediated regulation of exosome secretion had promising regenerative potential in tissue repair. To further evaluate these effects in vivo, we next investigated the therapeutic efficacy of TEAD1 delivered via an adeno‐associated virus (AAV) vector in a murine wound healing model.

**FIGURE 4:**
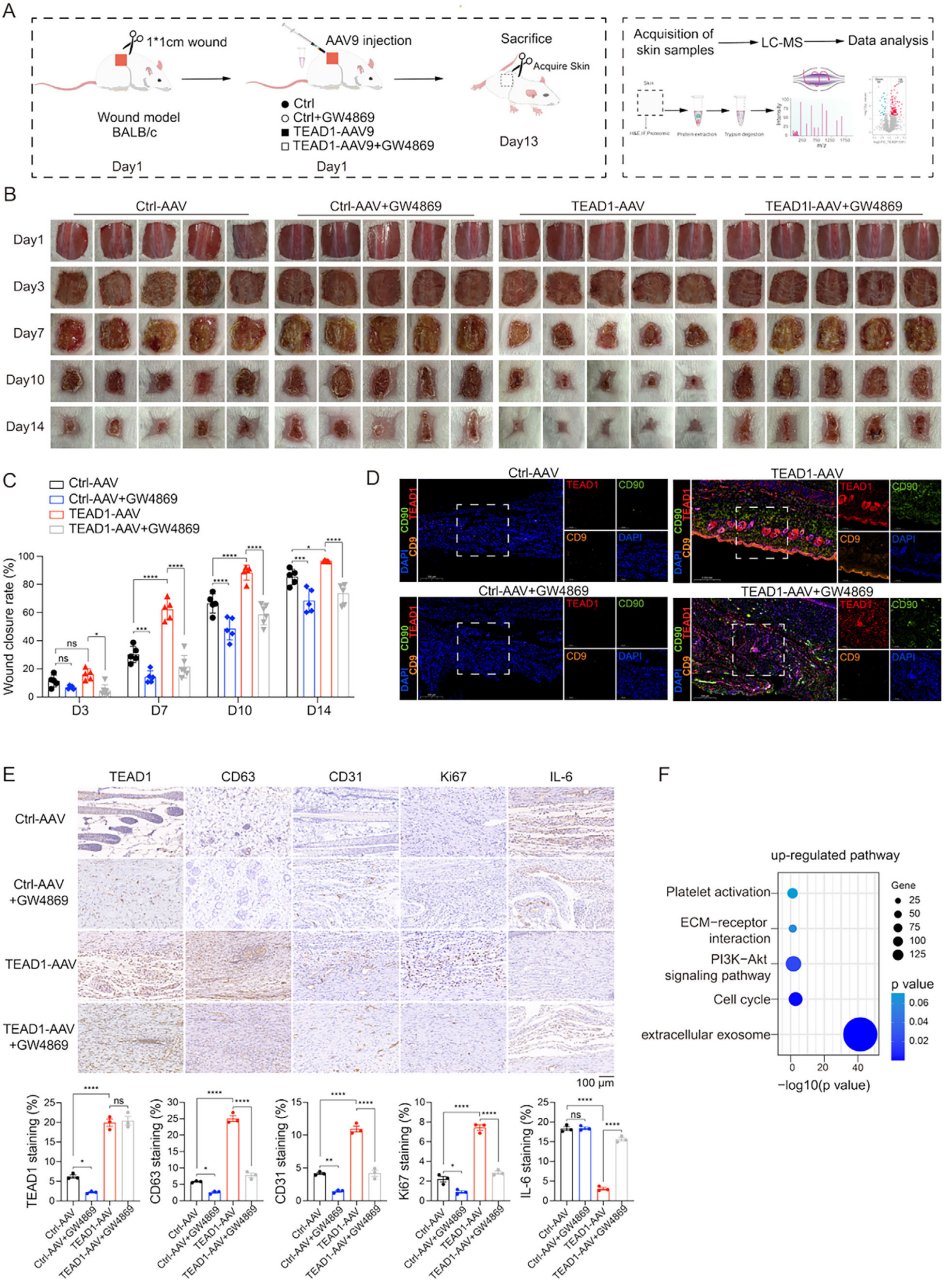
TEAD1‐AAV gene therapy enhances skin wound healing by promoting exosome secretion. (A) Establishment of 10 mm full‐thickness dermal dorsal defects in BALB/c mice and their corresponding proteomic analysis. (B,C) Images of the wound healing process of mice with different treatments (*n* = 5 per group, mean ± SD). (D) IF was performed to assess the expression of TEAD1, CD90, and CD9 in wound samples treated with Ctrl‐AAV, Ctrl‐AAV+GW4869, TEAD1‐AAV, or TEAD1‐AAV+GW4869 at day 14, scale bar, 200 µm. (E) IHC was performed to assess the expression of TEAD1, CD63, CD31, Ki67, and IL‐6 in wound samples treated with Ctrl‐AAV, Ctrl‐AAV+GW4869, TEAD1‐AAV, or TEAD1‐AAV+GW4869 at day 14, scale bar, 100 µm (*n* = 3 per group, mean ± SD). (F) Up‐regulated signaling pathways in the TEAD1‐AAV group. Differences among the groups were determined with one‐way ANOVA with Tukey's posttest. Data were considered statistically significant when *p* < 0.05 (∗), *p* < 0.01 (∗∗), *p* < 0.001 (∗∗∗), and *p* < 0.0001 (∗∗∗∗) versus the indicated group.

To assess the therapeutic effect of TEAD1‐AAV on wound healing and determine whether its regenerative effects depend on exosome secretion, we established 10 mm full‐thickness dermal dorsal defects in BALB/c mice (Figure 4A). The mice were randomly assigned to receive either TEAD1‐AAV, Ctrl‐AAV, TEAD1‐AAV+GW4869, or Ctrl‐AAV+GW4869 treatment. GW4869 is a well‐established pharmacological inhibitor of exosome biogenesis and release; it functions as a selective, noncompetitive inhibitor of neutral sphingomyelinase, thereby blocking ceramide‐dependent exosome formation in vivo [34]. Immunofluorescence (IF) staining for the exosomal marker CD9 demonstrated that GW4869 treatment markedly reduced exosome abundance in wound tissues, confirming effective inhibition of exosome secretion in vivo. IF and IHC analyses further confirmed successful overexpression of TEAD1 in the wound tissue of the TEAD1‐AAV and TEAD1‐AAV+GW4869 group, which was further validated by Western blot analysis (Figure 4D,E; Figure S9A,B). Notably, the MSC marker CD90 was also upregulated in the TEAD1‐AAV group compared with the Ctrl‐AAV group, supporting TEAD1's role in enhancing exosome‐mediated regenerative processes (Figure 4D). We next assessed the wound healing phenotype. Images of the wound were captured on days 1, 3, 7, 10, and 14, and body weights were continuously monitored throughout the healing process (Figure 4B,C). No significant differences were observed among the four groups, indicating that TEAD1‐AAV had no adverse impact on general health or systemic function (Figure S9C). TEAD1‐AAV significantly accelerated wound healing, and inhibition of exosome release using an exosome inhibitor reversed the therapeutic effect of TEAD1‐AAV, with clear differences emerging by day 7. Wound closure reached 68.26 ± 2.9% in the TEAD1‐AAV group, whereas Ctrl‐AAV and Ctrl‐AAV+GW4869 groups showed only 21.5 ± 1.9% and 14.3 ± 1.9% closure, respectively. Importantly, co‐administration of GW4869 with TEAD1‐AAV substantially attenuated healing, resulting in 21.5 ± 3.3% wound closure by day 7. By day 10, the TEAD1‐AAV group achieved a wound closure rate of 88.31 ± 2.4%, significantly higher than the 66.49 ± 3.0% observed in the Ctrl‐AAV group and the 58.49 ± 2.9% in the TEAD1‐AAV+GW4869 (Figure 4B,C), demonstrating both the potent reparative capacity of TEAD1 and the critical requirement of exosome secretion for its therapeutic effects. To further assess the underlying biological changes, wound tissues were collected for IHC analysis. IHC revealed that the TEAD1‐AAV group exhibited significantly increased expression of CD63, an exosomal marker, indicating enhanced exosome production in vivo. This was accompanied by a significant upregulation of CD31, a marker of vascular endothelial cells, reflecting enhanced angiogenesis during wound healing. Additionally, Ki67, an important marker of cell proliferation, was significantly upregulated in wound samples from the TEAD1‐AAV group compared to the Ctrl‐AAV group, whereas the pro‐inflammatory cytokine IL‐6 was reduced following TEAD1‐AAV treatment (Figure 4E). These results demonstrated that TEAD1 accelerated wound healing in mice by promoting exosome secretion, enhancing cell proliferation and angiogenesis, and reducing inflammatory responses. In contrast, TEAD1‐AAV+GW4869 significantly reduced CD63, CD31, and Ki67 expression, and elevated IL‐6 levels, indicating that inhibition of exosome secretion suppressed TEAD1‐driven angiogenic and proliferative responses, and extended the inflammatory response.

To better understand how TEAD1 facilitated wound healing, we performed proteomic analysis of regenerated skin tissue collected from mice treated with TEAD1‐AAV or Ctrl‐AAV. The proteomic profiling identified 144 DEPs between the TEAD1‐AAV and Ctrl‐AAV groups (Wilcoxon rank‐sum test, *p* < 0.05, FC > 2), including 417 upregulated and 29 downregulated proteins in the TEAD1‐AAV group (Figure S9D,E and Table S4a,b). Pathway enrichment analysis revealed that upregulated proteins in the TEAD1‐AAV group were mainly enriched in pathways such as platelet activation, ECM‐receptor interaction, PI3K‐Akt signaling pathway, cell cycle, and extracellular exosome (Figure 4F; Figure S9F). These pathways play pivotal roles in coordinating wound repair, as platelet activation initiates hemostasis and early inflammatory responses, ECM‐receptor interactions regulate cell adhesion and migration, PI3K‐Akt signaling promotes cell survival and angiogenesis, and cell cycle progression drives tissue regeneration.

These findings suggested that TEAD1 enhanced wound healing by orchestrating a multifaceted regenerative response involving exosome‐mediated signaling, vascular remodeling, and cellular proliferation.

### 2.5 TEAD1‐AAV Reversed Chronic Wounds in Type 1 and Type 2 Diabetes Through Enhanced Exosome Secretion

Diabetes mellitus was a rapidly growing global health concern, with an estimated 828 million adults affected worldwide in 2022, a dramatic increase from 1990 [35]. Among its complications, diabetic wounds represented a major clinical challenge due to delayed healing and high risk of infection and amputation [36]. Wound healing in both Type 1 Diabetes Mellitus (T1DM) and Type 2 Diabetes Mellitus (T2DM) is characterized by compromised angiogenesis and diminished collagen synthesis, both of which contribute significantly to the delayed closure of wounds.

To determine whether TEAD1‐AAV gene therapy improves chronic wound healing in both T1DM and T2DM, we established clinically relevant diabetic models. First, a streptozotocin (STZ)‐induced T1DM mouse model was established [37]. T1DM mice were characterized by elevated blood glucose levels, increased food and water intake, and decreased body weight. The fasting blood glucose (FBG) of the STZ group was stable soon after the injection of STZ, but it significantly increased on the fifth day compared to the wild‐type (WT). On the 10th day, the mean FBG was over 16 mmol/L, which met the blood glucose level requirement for diabetes (Figure S9G). The body weight of the STZ group was significantly decreased on the fifth and 10th day in comparison with that of the WT (Figure S9H). The comprehensive dataset validated the successful establishment of the T1DM model.

**FIGURE 5:**
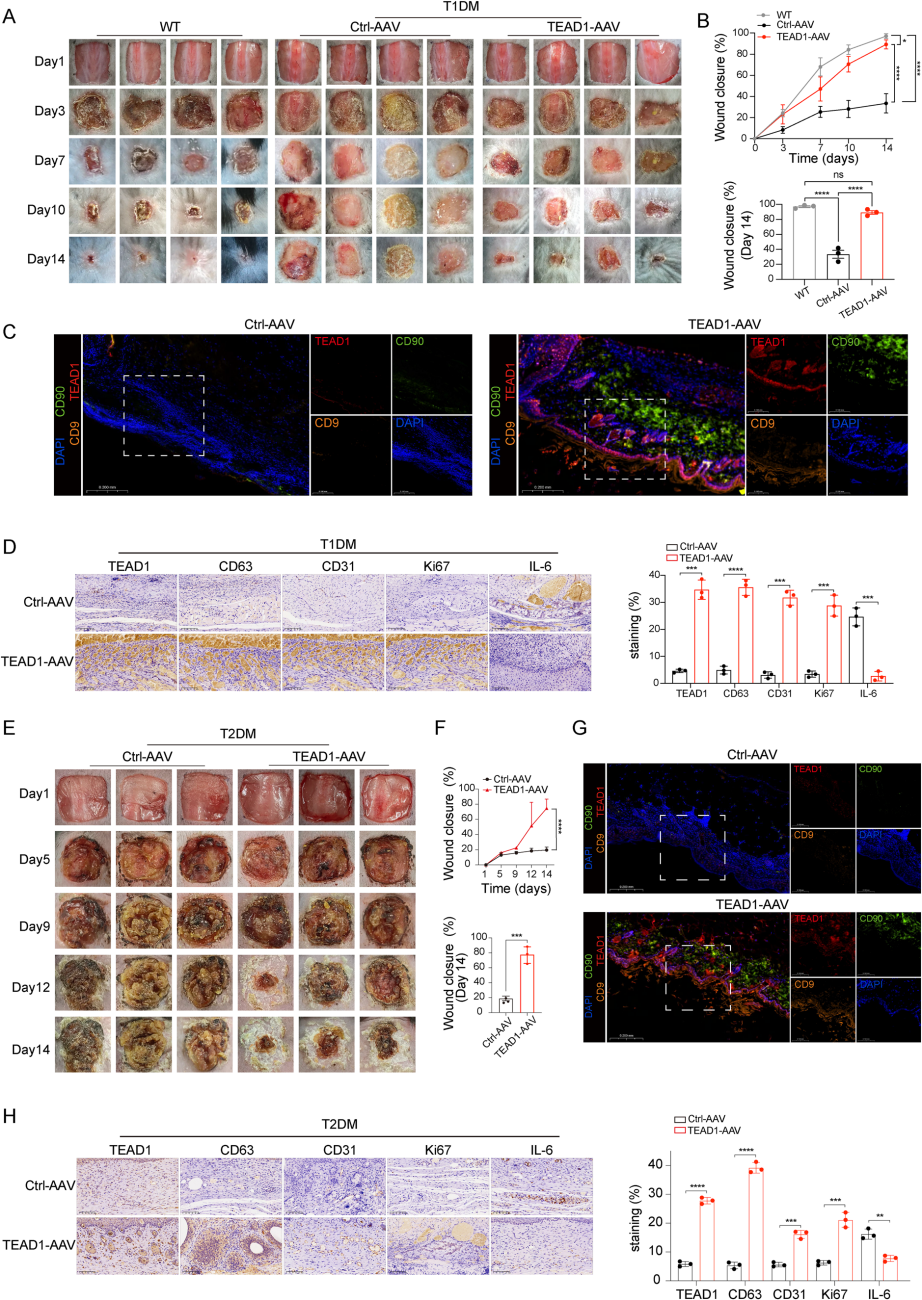
TEAD1‐AAV reversed chronic wounds in Type 1 and Type 2 diabetes through enhanced exosome secretion. (A) Images of the wound healing process of WT and T1DM mice with different treatments (*n* = 4 per group). (B) Wound closure rates of WT and T1DM mice with different treatments (*n* = 3 per group, mean ± SD). (C) IF staining was performed to assess the expression of TEAD1, CD90, and CD9 in T1DM mice treated with TEAD1‐AAV or Ctrl‐AAV at day 14, scale bar, 200 µm. (D) IHC staining was performed to assess the expression of TEAD1, CD63, CD31, Ki67, and IL‐6 in T1DM mice treated with TEAD1‐AAV or Ctrl‐AAV at day 14, scale bar, 100 µm (*n* = 3 per group, mean ± SD). (E) Images of the wound healing process of T2DM mice with different treatments (*n* = 3 per group). (F) Wound closure rates of T2DM mice with different treatments (*n* = 3 per group, mean ± SD). (G) IF staining was performed to assess the expression of TEAD1, CD90, and CD9 in T2DM mice treated with TEAD1‐AAV or Ctrl‐AAV at day 14, scale bar, 200 µm. (H) IHC staining was performed to assess the expression of TEAD1, CD63, CD31, Ki67, and IL‐6 in T2DM mice treated with TEAD1‐AAV or Ctrl‐AAV at day 14, scale bar, 100 µm (*n* = 3 per group, mean ± SD). Differences among the groups were determined with one‐way ANOVA with Tukey's posttest and Student's two‐tailed t‐test. Data were considered statistically significant when *p* < 0.05 (∗), *p* < 0.01 (∗∗), *p* < 0.001 (∗∗∗), and *p* < 0.0001 (∗∗∗∗) versus the indicated group.

To evaluate the therapeutic potential of TEAD1‐AAV, wounds were generated in T1DM mice and treated with either TEAD1‐AAV or Ctrl‐AAV. IF and IHC staining confirmed successful overexpression of TEAD1 in the TEAD1‐AAV group (Figure 5C,D). Notably, IF analysis revealed significantly increased expression of the exosomal marker CD9 and the MSC marker CD90 in the TEAD1‐AAV group compared with the Ctrl‐AAV group (Figure 5C), indicating enhanced exosome secretion and MSC‐related activity in vivo. These results suggested that TEAD1‐AAV not only stimulated exosome production but may also promoted regenerative signaling pathways associated with MSC function, thereby contributing to tissue repair in T1DM. Optical images were captured on days 1, 3, 7, 10, and 14 post‐injury in WT and T1DM mice treated with either TEAD1‐AAV or Ctrl‐AAV (Figure 5A). By day 7 post‐injury, a significant divergence in wound healing became apparent, with the TEAD1‐AAV group exhibiting a wound closure rate of 47.1 ± 6.6%, compared to only 25.4 ± 3.0% in the Ctrl‐AAV group. This difference further intensified over time, reaching 89.3 ± 2.5% closure in the TEAD1‐AAV group by day 14, while the Ctrl‐AAV group achieved only 33.4 ± 5.3% (Figure 5A,B). These results underscored the impaired healing dynamics in T1DM and demonstrated the potent therapeutic effect of TEAD1‐AAV in promoting tissue repair. IHC staining further revealed elevated expression of CD63 (an exosome membrane marker), CD31 (a marker of endothelial cells and angiogenesis), and Ki67 (a marker of cellular proliferation) in the TEAD1‐AAV group compared to the Ctrl‐AAV group (Figure 5D), indicating enhanced exosome release, increased neovascularization, and accelerated cell proliferation during wound healing. Conversely, the inflammatory cytokine IL‐6 was significantly reduced in the TEAD1‐AAV group, reflecting a suppressed inflammatory response, which is crucial for resolving chronic inflammation and enabling tissue regeneration.

Given the promising therapeutic efficacy of TEAD1‐AAV in promoting wound repair in T1DM, we sought to determine whether this benefit could be extended to T2DM, a more prevalent and complex form of diabetes that accounts for over 90% of all diabetes cases worldwide [38]. Demonstrating the efficacy of TEAD1‐AAV in a T2DM mouse wound model would further strengthen its translational relevance for diabetic wound therapy across a broader clinical spectrum. We next evaluated the therapeutic efficacy of TEAD1‐AAV in a T2DM wound model established in db/db mice. Consistent with the findings in the T1DM model, IF and IHC staining confirmed successful overexpression of TEAD1 in the TEAD1‐AAV group (Figure 5G‐H). IF analysis also showed markedly increased expression of CD9 and CD90 in TEAD1‐AAV‐treated wounds compared to those treated with Ctrl‐AAV (Figure 5G), suggesting enhanced exosome secretion and MSC‐associated activity in T2DM wounds. Representative optical images of wound sites were captured on days 1, 3, 7, 10, and 14 post‐injury in T2DM mice treated with TEAD1‐AAV or Ctrl‐AAV (Figure 5E). By day 14, the TEAD1‐AAV group exhibited a substantially improved healing response, achieving an average wound closure rate of 77.1 ± 6.3%, compared to only 18.6 ± 1.8% in the Ctrl‐AAV group (Figure 5E,F). These findings further underscore the impaired healing capacity typical of T2DM wounds and demonstrate that TEAD1‐AAV effectively enhanced tissue repair even in this more metabolically compromised diabetic context. Furthermore, IHC staining revealed increased expression of CD63, CD31, and Ki67 in the TEAD1‐AAV group compared to the Ctrl‐AAV group (Figure 5H), indicating enhanced exosome secretion, angiogenesis, and cellular proliferation. Conversely, IL‐6 expression was significantly reduced in the TEAD1‐AAV group, suggesting a decrease in local inflammation, an essential component for resolving chronic wounds and promoting tissue repair.

To gain further insight into how TEAD1 promoted wound healing, we performed proteomic analyses of regenerated skin tissues from T1DM and T2DM mouse models treated with either TEAD1‐AAV or Ctrl‐AAV. In the T1DM model, a total of 314 proteins were differentially expressed between the TEAD1‐AAV and Ctrl‐AAV groups (Wilcoxon rank‐sum test, *p* < 0.05, FC > 2), including 67 upregulated and 247 downregulated in the TEAD1‐AAV group (Figure S9I and Table S4c,d). Pathway enrichment analysis indicated that proteins with higher expression in the TEAD1‐AAV group were significantly enriched in wound healing‐related pathways, including extracellular exosome, collagen‐containing extracellular matrix, structural components of the skin epidermis, and extracellular matrix organization (Figure S9J,K). Similarly, in the T2DM model, 137 proteins were differentially expressed between the TEAD1‐AAV and Ctrl‐AAV groups, with 73 upregulated and 64 downregulated in the TEAD1‐AAV group (Wilcoxon rank‐sum test, *p* < 0.05, FC > 2, Figure S9L and Table S4e,f). Enrichment analysis showed that the upregulated proteins in the TEAD1‐AAV group were mainly involved in vesicle‐mediated transport, cell adhesion, wound healing, actin cytoskeleton organization, and cell migration (Figure S9M,N). These findings suggested that TEAD1 enhanced wound repair by promoting exosome secretion and activating diverse regenerative pathways involved in extracellular matrix remodeling, cellular motility, and tissue regeneration.

In summary, TEAD1‐AAV gene therapy significantly enhanced exosome secretion, stimulated endothelial cell proliferation and angiogenesis, and effectively promoted skin wound healing in both T1DM and T2DM mouse models. These findings highlighted TEAD1‐AAV as a promising therapeutic strategy for the treatment of chronic wounds associated with diabetes.

### 2.6 TEAD1 Enhances BMSC‐Exos Secretion and Promotes Neurite Regeneration and Neural Repair

Our studies have shown that TEAD1 enhances exosome secretion by ADSCs, thereby promoting skin wound healing in diabetic mice. Notably, recent studies have shown that exosomes derived from BMSCs also exhibit remarkable potential in neural injury repair [39, 40]. Given that neural injury may lead to irreversible, lifelong functional impairments, there exists a more urgent clinical demand for neural repair strategies [41]. Therefore, based on the potent ability of TEAD1 to promote exosome secretion and its demonstrated efficacy in wound healing, we further investigated its therapeutic potential in more complex neural injuries.

**FIGURE 6:**
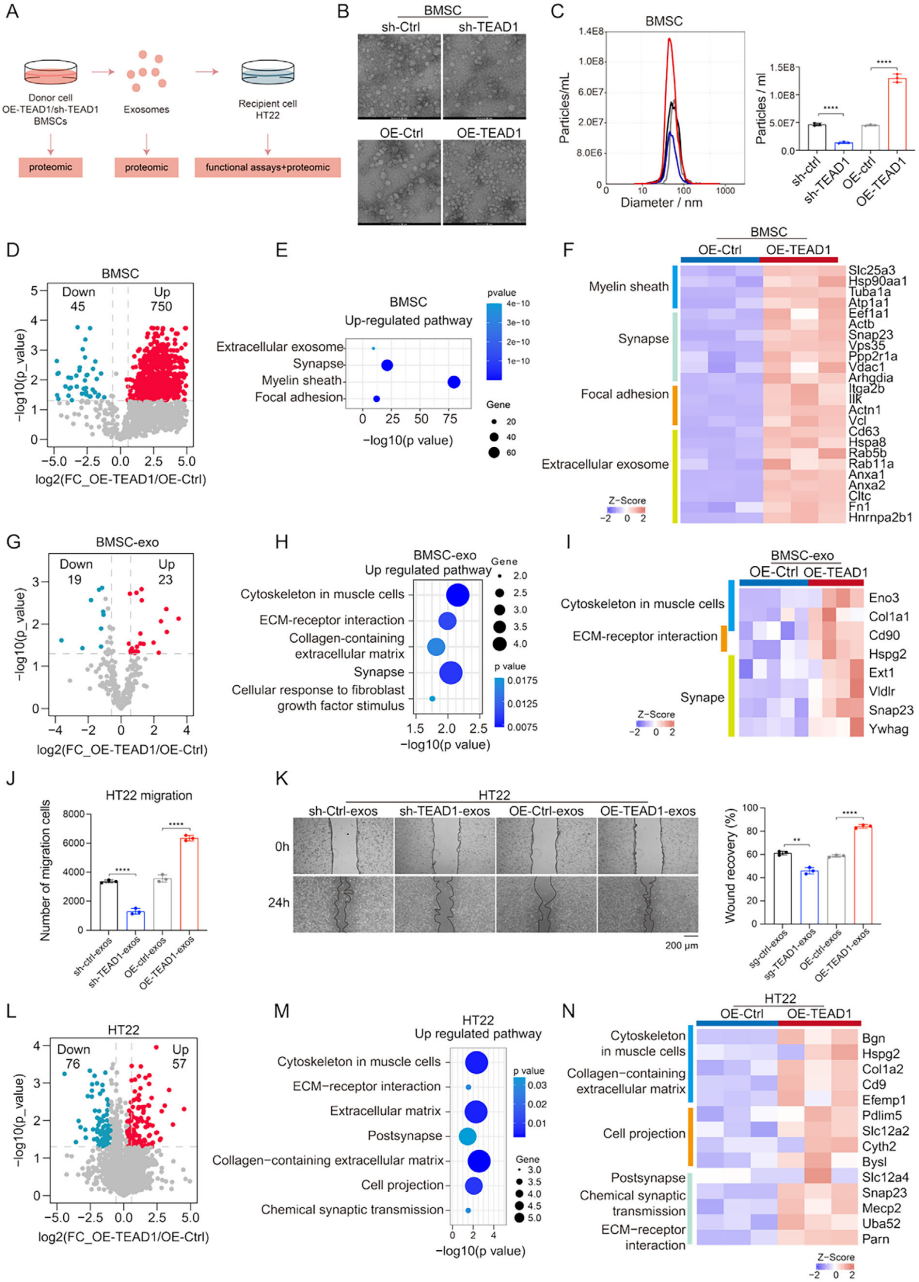
TEAD1 enhances BMSC‐Exos secretion and promotes protrusion regeneration and neural repair. (A) Experimental design for assessing exosome‐mediated effects of TEAD1 modulation in BMSCs. (B) TEM images of exosomes after TEAD1 knockdown and overexpression in BMSCs. Scale bar = 50 nm. (C) Statistics of exosome size and particle number after TEAD1 knockdown and overexpression in BMSCs (*n* = 3 per group, mean ± SD). (D) Differential protein expression in BMSCs upon TEAD1 overexpression. Red indicates up‐regulated proteins, while blue indicates down‐regulated ones. (E) Up‐regulated signaling pathways in BMSCs overexpressing TEAD1. (F) The heatmap showing the DEPs in the OE‐TEAD1 and the OE‐Ctrl groups, the expression of proteins participating in the myelin sheath, synapse, focal adhesion, and extracellular exosome in OE‐TEAD1 and OE‐Ctrl cells is shown (*n* = 3 per group). (G) Differential protein expression in OE‐TEAD1 BMSCs‐exos or OE‐Ctrl BMSCs‐exos groups. Red indicates up‐regulated proteins, while blue indicates down‐regulated ones. (H) Up‐regulated signaling pathways in OE‐TEAD1 BMSCs‐exos group. (I) The heatmap showing the DEPs in OE‐TEAD1 BMSCs‐exos and OE‐Ctrl BMSCs‐exos groups, the expression of proteins participating in the cytoskeleton in muscle cells, ECM‐receptor interaction, and synapse in OE‐TEAD1 BMSCs‐exos (*n* = 4) and OE‐Ctrl BMSCs‐exos (*n* = 5) is shown. (J) Transwell assays demonstrated the effects of exosomes from different sources on the migration of HT22 cells (*n* = 3 per group, mean ± SD). (K) The scratch assay results of HT22 cells treated with exosomes from different sources, scale bar, 200 µm (*n* = 3 per group, mean ± SD). (L) Differential protein expression in HT22 cells treated with OE‐TEAD1 BMSCs‐exos or OE‐Ctrl BMSCs‐exos. Red indicates up‐regulated proteins, while blue indicates down‐regulated ones. (M) Up‐regulated signaling pathways in HT22 cells treated with OE‐TEAD1 BMSCs‐exos. (N) The heatmap showing the DEPs in HT22 cells treated with OE‐TEAD1 BMSCs‐exos or OE‐Ctrl BMSCs‐exos, the expression of proteins participating in the cytoskeleton in muscle cells, collagen‐containing extracellular matrix, cell projection, postsynapse, chemical synaptic transmission, and ECM‐receptor interaction in HT22 cells is shown (*n* = 3 per group). Differences among the groups were determined with one‐way ANOVA with Tukey's posttest and Student's two‐tailed t‐test. Data were considered statistically significant when *p* < 0.01 (∗∗) and *p* < 0.0001 (∗∗∗∗) versus the indicated group.

To investigate whether TEAD1‐mediated enhancement of exosome biogenesis contributed to neural injury repair, we isolated primary mouse BMSCs and established TEAD1 overexpression (OE‐TEAD1) and TEAD1 knockdown (sh‐TEAD1) BMSC lines. The efficiency of TEAD1 overexpression and knockdown was confirmed at both the mRNA and protein levels (Figure S10A,B). Subsequently, exosomes were isolated and characterized from these cell lines for further analysis (Figure 6A,B; Figure S10C). Quantitative analysis showed that OE‐TEAD1 significantly enhanced BMSC‐derived exosome (BMSC‐Exos) secretion compared to the control group, whereas sh‐TEAD1 markedly reduced exosome secretion (Figure 6C).

To elucidate the regulatory role of TEAD1 in exosome secretion by BMSCs and its potential impact on neural repair, we performed proteomic analysis on OE‐TEAD1 and control BMSC lines. The proteomic profiling identified DEPs between the OE‐TEAD1 and the control groups in BMSC lines (Figure 6D; Table S5a,b). Notably, proteins involved in exosome biogenesis and secretion, such as SNAP23 and CD9, were significantly upregulated in the OE‐TEAD1 group. Compared with the control group, the upregulated proteins in the OE‐TEAD1 group were mainly enriched in pathways related to extracellular exosomes, synapses, myelin sheath, and focal adhesion (Figure 6E,F). These findings suggested that TEAD1 enhanced BMSC‐Exo secretion and promoted neural repair by upregulating proteins involved in synaptic growth and cell adhesion.

To assess whether TEAD1 influenced exosomal protein content, we performed proteomic analysis of exosomes isolated from OE‐TEAD1 and control BMSC cell lines. Among 639 identified exosomal proteins, only 42 (19 upregulated, 23 downregulated; approximately 6% of the total proteins) were differentially expressed (Wilcoxon rank‐sum test, *p* < 0.05, FC > 2; Figure 6G; Table S5c,d). Pathway enrichment analysis of the upregulated proteins in the TEAD1‐AAV group revealed significant activation of pathways related to exosome biogenesis and secretion, as well as neuroregeneration and tissue remodeling, including cytoskeleton in muscle cells, ECM‐receptor interaction, and synapse‐related signaling pathways (Figure 6H,I). These findings indicated that although TEAD1 induced only minor global changes in the exosomal proteome of BMSCs, it selectively upregulated proteins associated with exosome biogenesis, neuroregeneration, and tissue remodeling, thereby enhancing the regenerative potential of BMSC‐Exos.

To investigate the role of TEAD1‐regulated BMSC‐Exos in promoting neuronal migration and regeneration, we examined their effects on neural cells in vitro. Specifically, exosomes from OE‐TEAD1 or sh‐TEAD1 BMSCs were co‐cultured with HT22 cells (mouse hippocampal neuronal cell line), and their effects on cell migration were evaluated (Figure S10D). The results showed that OE‐TEAD1 BMSC‐Exos significantly enhanced the migratory capacity of HT22 cells compared to control BMSC‐Exos, whereas sh‐TEAD1 BMSC‐Exos did not exert a promotive effect on neuronal migration (Figure 6J,K). These findings suggested that OE‐TEAD1 BMSC‐Exos enhanced the neuroregenerative potential of BMSCs by promoting neuronal migration, compared with control BMSC‐Exos. Accumulating evidence has suggested that exosomes repair nerve damage by activating regenerative processes within neurons and promoting axonal growth [42].

To explore how OE‐TEAD1 BMSC‐Exos contributed to nerve regeneration, we performed proteomic analysis of recipient HT22 cells. The proteomic profiling identified DEPs between cells treated with OE‐TEAD1 BMSC‐Exos and those treated with control BMSC‐Exos (Wilcoxon rank‐sum test, *p* < 0.05, fold change > 2; Figure 6L; Table S5e,f). Pathway enrichment analysis revealed that, compared to the control group, upregulated proteins in HT22 cells treated with OE‐TEAD1 BMSC‐Exos were primarily involved in cytoskeleton in muscle cells, ECM‐receptor interaction, extracellular matrix, postsynapse, collagen‐containing extracellular matrix, cell projection, and chemical synaptic transmission (Figure 6M,N). These findings suggested that exosomes derived from OE‐TEAD1 BMSCs primarily activate proteins related to cytoskeletal remodeling and synaptic regeneration, thereby facilitating neural cell repair.

Together, TEAD1 facilitated BMSC‐Exo biogenesis and secretion by upregulating key exosomal proteins, which in turn enhanced neuronal migration and activated cytoskeletal and synaptic remodeling pathways in recipient neural cells, thereby underscoring its mechanistic role and therapeutic potential in promoting neural regeneration and repair.

### 2.7 TEAD1‐AAV Therapy Enhances Exosome Secretion and Facilitates Functional Recovery After SCI

SCI is a devastating condition of the central nervous system, often resulting in permanent sensorimotor deficits and a sharp decline in quality of life. Despite decades of research, effective therapeutic strategies for SCI remain elusive due to the limited regenerative capacity of adult spinal neurons and the formation of a hostile microenvironment post‐injury, including glial scarring, chronic inflammation, and disrupted axon guidance [43]. Moreover, existing research has provided substantial evidence supporting the contribution of exosomes to the enhancement of functional recovery in mice following SCI [44]. Therefore, we further investigated the therapeutic efficacy of TEAD1‐AAV in SCI repair.

**FIGURE 7:**
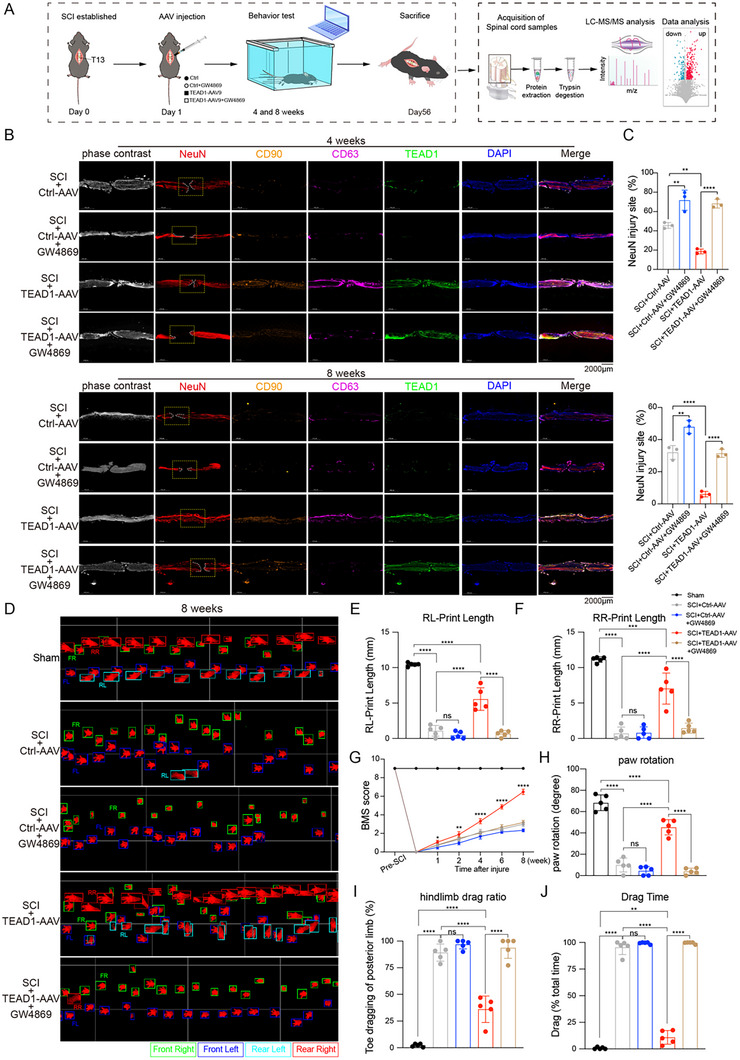
TEAD1‐AAV therapy enhances exosome secretion and facilitates functional recovery after SCI. (A) Establishment of SCI in C57 mice and their corresponding proteomic analysis. (B,C) IF staining at weeks 4 and 8 after SCI (B), and the NeuN injury ratio (C). NeuN (red), TEAD1 (green), CD90 (orange), CD63 (purple), and DAPI (blue); scale bar = 2 mm, (*n* = 3 per group, mean ± SD). (D) Typical diagrams of TreadScan test in Sham, Ctrl‐AAV, Ctrl‐AAV+GW4869, TEAD1‐AAV, and TEAD1‐AAV+GW4869 groups of mice at week 8 post‐injury. (E,F) Print length of the left (E) and right (F) rear limbs in Sham, Ctrl‐AAV, Ctrl‐AAV+GW4869, TEAD1‐AAV, and TEAD1‐AAV+GW4869 groups of mice at week 8 post‐injury (*n* = 5 per group, mean ± SD). (G) Graph indicates the locomotor BMS scores in Sham, Ctrl‐AAV, Ctrl‐AAV+GW4869, TEAD1‐AAV, and TEAD1‐AAV+GW4869 groups (*n* = 5 per group, mean ± SD). (H) Paw rotation in Sham, Ctrl‐AAV, Ctrl‐AAV+GW4869, TEAD1‐AAV, and TEAD1‐AAV+GW4869 groups of mice at week 8 post‐injury (*n* = 5 per group, mean ± SD). (I) Hindlimb drag ratio in Sham, Ctrl‐AAV, Ctrl‐AAV+GW4869, TEAD1‐AAV, and TEAD1‐AAV+GW4869 groups of mice at week 8 post‐injury (*n* = 5 per group, mean ± SD). (J) Drag Time in Sham, Ctrl‐AAV, Ctrl‐AAV+GW4869, TEAD1‐AAV, and TEAD1‐AAV+GW4869 groups of mice at week 8 post‐injury (*n* = 5 per group, mean ± SD). Differences among the groups were determined with one‐way ANOVA with Tukey's posttest. For BMS behavioral assessments, data were analyzed using two‐way repeated‐measures ANOVA. Data were considered statistically significant when *p* < 0.01 (∗∗), *p* < 0.001 (∗∗∗), and *p* < 0.0001 (∗∗∗∗) versus the indicated group.

To investigate the therapeutic potential of TEAD1‐AAV in SCI and determine whether its regenerative effects depend on exosome secretion, we established a complete transection model at the thoracic vertebra T13 in C57 mice [45]. The mice received local injections of TEAD1‐AAV, Ctrl‐AAV, TEAD1‐AAV+GW4869, or Ctrl‐AAV+GW4869 at the lesion site one day post‐SCI (Figure 7A). IF analysis confirmed successful transgene expression of TEAD1 in spinal cord tissue (Figure 7B). IF staining for the exosomal marker CD63 demonstrated that TEAD1 significantly increased exosome secretion, while GW4869 treatment markedly reduced exosome abundance in wound tissues. Moreover, neuronal markers NeuN and MSC marker CD90 showed enhanced neuronal regeneration in the TEAD1‐AAV group compared to the Ctrl‐AAV group. However, co‐administration of GW4869 significantly diminished CD63 and NeuN expression despite sustained TEAD1 overexpression, demonstrating that inhibition of exosome release effectively blocked TEAD1‐driven neuroregeneration at the lesion site (Figure 7B,C). Importantly, these morphological improvements translated into significant functional recovery over the 8‐week post‐SCI period. Gait analysis using the automated treadmill‐based TreadScan system [46] revealed that TEAD1‐AAV‐treated mice exhibited markedly enhanced locomotor performance, characterized by increased stride length and improved hindlimb coordination at 8 weeks post‐injury (Figure 7D,F). In contrast, TEAD1‐AAV+GW4869 mice failed to exhibit meaningful functional improvement and instead resembled Ctrl‐AAV–treated mice, which maintained persistent hindlimb dragging, underscoring the essential role of TEAD1‐driven exosome secretion in mediating functional motor recovery. Consistently, the TEAD1‐AAV group demonstrated significantly higher Basso mouse scale (BMS) scores [47] compared to controls at 1, 2, 4, 6, and 8 weeks post‐injury. By the 8 week, TEAD1‐AAV‐treated mice reached a median BMS score of 6.5, indicative of frequent plantar stepping with occasional coordination, whereas control mice plateaued at a score of 3.0, reflecting predominant dorsal stepping without plantar support. In contrast, TEAD1‐AAV+GW4869 mice plateaued at low BMS scores at 3.2, similar to Ctrl‐AAV groups, reflecting poor stepping ability and lack of coordinated hindlimb engagement (Figure 7G), further confirming that restoration of motor function requires TEAD1‐induced exosome release. Furthermore, paw rotation degree data obtained from the footprint analysis revealed comparable patterns. The mean paw rotation degree that the mice in the TEAD1‐AAV group could bear was 45.29°, which was much higher than that in the Ctrl‐AAV (9.98°), TEAD1‐AAV+GW4869 (4.07°), and Ctrl‐AAV+GW4869 (4.44°) groups (Figure 7H). Furthermore, hindlimb dragging data obtained from the footprint analysis showed that mice in the Sham group exhibited no hindlimb dragging, whereas mice in the Ctrl‐AAV group exhibited a significantly higher hindlimb dragging ratio (89.24%). In contrast, TEAD1‐AAV treatment significantly alleviated post‐SCI motor impairment, as evidenced by a substantial reduction in the hindlimb dragging ratio to 36.1%. Notably, inhibition of exosome secretion with GW4869 abolished this therapeutic benefit, with TEAD1‐AAV+GW4869–treated mice exhibiting a hindlimb dragging ratio of 93.82%, which was comparable to that observed in the Ctrl‐AAV group, indicating no functional improvement (Figure 7I,J). These improvements were abolished by GW4869, further confirming that restoration of motor function requires TEAD1‐induced exosome release. These results indicate that TEAD1‐AAV treatment substantially promotes exosome secretion and neuronal regeneration, thereby facilitating robust locomotor and functional recovery in a complete SCI model.

To better understand the therapeutic impact of TEAD1‐AAV on SCI, we performed proteomic analysis of spinal cord tissues collected from mice treated with either TEAD1‐AAV or Ctrl‐AAV. A total of 476 proteins were differentially expressed between the TEAD1‐AAV and Ctrl‐AAV groups (Wilcoxon rank‐sum test, *p* < 0.05, FC > 2), including 345 upregulated and 131 downregulated proteins in the TEAD1‐AAV group (Figure S11A and Table S6a). Enrichment analysis showed that the upregulated proteins in the TEAD1‐AAV group were predominantly associated with pathways involved in axon guidance, regenerative axon extension, synaptic cleft, extracellular exosome, and ECM‐receptor interaction (Figure S11B,C and Table S6b). These results suggested that TEAD1‐AAV promoted spinal cord repair by enhancing exosome secretion, which in turn activated multiple regenerative signaling pathways, particularly those associated with axonal growth, synaptic remodeling, and cell–matrix communication.

In summary, these results revealed a previously unrecognized role of TEAD1 in modulating exosome‐mediated neuroregeneration and highlighted its translational potential for SCI treatment.

## 3 Discussion

In this study, TEAD1 emerged as a novel and central transcription factor downstream of the Hippo pathway, playing a critical role in the regulation of exosome biogenesis. The Hippo pathway is a highly conserved signaling cascade that regulates organ size and tissue homeostasis by modulating cell proliferation and survival. As a major downstream effector, TEAD1 translates mechanical and biochemical cues into gene expression programs governing growth and regeneration [48], and dysregulation of this pathway has been associated with tumorigenesis and impaired tissue repair.

Using a lung cancer metastasis model, we observed that metastatic cells exhibited elevated TEAD1 expression accompanied by increased exosome secretion, suggesting that TEAD1 may modulate the cellular microenvironment through vesicular communication. Functional analyses demonstrated that TEAD1 directly upregulates key vesicular trafficking genes, including RAB11, CD9, and SNAP23, thereby promoting exosome biogenesis and release. Notably, other members of the TEAD transcription factor family, including TEAD2, TEAD3, and TEAD4, were also capable of enhancing exosome secretion, their effects were comparatively less pronounced than those of TEAD1. TEAD1 exerts a dominant role in coordinating exosome biogenesis. The precise and potentially context‐dependent contributions of TEAD2, TEAD3, and TEAD4 to exosome regulation remain to be fully elucidated and merit further investigation. Although other TFs, such as STAT1, exhibited modest effects on exosome output, no alterations in STAT‐related TFRE signals were observed across different cell generations, suggesting that their influence on exosome secretion is likely indirect. Importantly, these findings offer a new perspective: the regulation of exosome secretion, mediated by Hippo–TEAD1 signaling, may be a fundamental mechanism by which organ size and tissue remodeling are controlled. Exosomes may serve as systemic conveyors of growth‐regulatory signals, with TEAD1 acting as a central transcriptional switch in this network. Consistent with this view, we found that TEAD1 enhances tissue repair and regeneration in models of diabetic wound healing and SCI, further supporting its therapeutic potential.

Previous studies have explored multiple strategies to enhance exosome production, including physical stimulation (e.g., hypoxia, shear stress), chemical treatments (e.g., calcium ionophores, small molecules), and genetic modifications targeting components of the exosome biogenesis pathway, such as overexpression of Rab GTPases or ESCRT machinery proteins [2, 16]. While these methods can elevate exosome yield, they often lack specificity and may disrupt cellular homeostasis. In contrast, our approach using TEAD1‐AAV offers a more targeted and potentially safer method for enhancing exosome biogenesis. TEAD1 can upregulate CD9 and SNAP23 genes associated with vesicle trafficking and exosome release, resulting in robust exosome production while maintaining cellular integrity. A major advantage of our TEAD1‐AAV approach is its ability to sustainably and intrinsically enhance exosome production in vivo, without compromising cell viability or requiring exogenous stimuli. Compared to conventional exosome‐based therapies, this strategy eliminates the need for labor‐intensive exosome isolation and ex vivo manipulation. Importantly, AAV vectors have been extensively validated for their safety, low immunogenicity, and broad tropism across tissues, making this approach both clinically translatable and scalable [49]. Furthermore, TEAD1 upregulation may promote exosome secretion as part of a coordinated cellular program, potentially leading to exosomes with more consistent and functional cargo profiles. This may be particularly beneficial for therapeutic applications where exosome quality and bioactivity are critical.

As the largest organ of the human body, the skin consists of diverse cell types that collectively maintain tissue homeostasis [50, 51]. Wound healing is vital for restoring epidermal integrity after injury and typically proceeds through four stages: hemostasis, inflammation, proliferation, and remodeling [52, 53]. However, this process is often impaired in patients with metabolic or inflammatory conditions, particularly diabetes [54, 55]. Globally, hundreds of millions of individuals live with diabetes, and about 10%–25% develop chronic wounds or diabetic foot ulcers [56], which have a 5‐year mortality rate of approximately 50%, rising to 80% after amputation [57]. Delayed healing in diabetic wounds is mainly attributed to hyperglycemia‐induced microvascular dysfunction, peripheral neuropathy, and persistent inflammation. Although current treatments such as debridement, grafting, dressings, and hyperbaric oxygen therapy are available, they are limited by high cost, long recovery time, and infection risk [58]. Therefore, new strategies for more effective treatments for wound healing, especially chronic wounds, are urgently needed for clinical application. Our study illustrates the role of TEAD1 in promoting exosome secretion from ADSCs and emphasizes its therapeutic benefit in promoting skin injury repair. In addition to characterizing TEAD1's mechanistic role, we further developed an AAV‑TEAD1 delivery approach that enables targeted and long‐lasting modulation of regenerative signaling in vivo, highlighting its potential as a clinically translatable strategy for regenerative medicine.

SCI is a devastating condition that often results in permanent paralysis and imposes a significant burden on individuals, families, and society [59]. Globally, SCI affects approximately 40–80 people per million each year [60]. It causes severe impairments in sensory, motor, and autonomic functions, often accompanied by complications such as motor deficits, gastric dysfunction, cardiac abnormalities, and bladder disorders [61]. Following the primary mechanical injury, secondary damage, including neuronal death, vascular rupture, disruption of the blood‐spinal cord barrier (BSCB), and inflammatory responses, further exacerbates tissue degeneration and hinders recovery [62]. Due to its debilitating effects on quality of life and survival, SCI remains a major clinical challenge, as effective therapies capable of restoring neurological function are still lacking. Exosomes derived from MSCs can cross the BSCB and promote neural repair by reducing neuronal apoptosis, enhancing vascular remodeling and neurogenesis, suppressing neuroinflammation, and supporting axonal regeneration. Our study highlights the role of TEAD1 in promoting exosome secretion from BMSCs and underscores its therapeutic benefits in facilitating SCI repair. In addition to revealing this mechanism, we employed an AAV‑mediated TEAD1 delivery strategy that enables convenient, localized, and sustained modulation of regenerative pathways in vivo, leveraging the minimal pathogenicity and long‑term expression features of AAV vectors that make them attractive for targeted gene delivery with reduced systemic exposure [63].

TEAD1 exhibits a context‐dependent dual role in both regeneration and tumorigenesis. Dysregulated YAP/TEAD signaling drives epithelial–mesenchymal transition, stemness, and metastasis in various cancers, including lung, breast, and liver malignancies. Conversely, TEAD1 can also act as a homeostatic regulator in normal tissues; its deletion in pancreatic β‐cells enhances proliferation, and reduced TEAD1 expression correlates with poor prognosis in certain tumors, such as renal cell carcinoma. These observations underscore that TEAD1's function is cell type– and context‐dependent, balancing proliferative and regenerative outputs. Notably, TEAD1 overexpression alone does not induce spontaneous tumorigenesis in vivo. Multiple transgenic and conditional TEAD1‐overexpressing mouse models in muscle and cardiac tissues have shown no evidence of neoplasia, even with long‐term expression. TEAD1's potential oncogenicity thus appears to depend on concurrent activation of YAP/TAZ or suppression of upstream Hippo regulators, rather than TEAD1 itself. Our findings demonstrated that TEAD1 enhanced exosome release, thereby promoting tissue regeneration, without altering the molecular composition of the exosomes. Furthermore, AAV‐mediated TEAD1 delivery achieves moderate, tissue‐restricted expression, offering an added safety margin. Together, these data support that TEAD1‐enhanced exosome production represents a biologically safe and controllable approach to stimulate regeneration while maintaining low tumorigenic risk. Future safety evaluation should focus on dose–response optimization, tissue‐specific expression systems, and long‐term toxicology assessments, particularly in highly regenerative organs. Engineered exosomes derived from TEAD1‐activated mesenchymal stem cells may also serve as a safer alternative to direct host TEAD1 activation, harnessing regenerative benefits while minimizing systemic exposure.

In summary, our findings demonstrate that TEAD1 functions as a crucial molecular switch regulating exosome secretion in various cell types. TEAD1 enhances exosome secretion by upregulating key proteins associated with exosome secretion, including RAB11, CD9, and SNAP23. This study reveals a novel role for TEAD1 in regulating exosome secretion and tissue regeneration, particularly in diabetic wound healing and SCI models. Moreover, TEAD1‐AAV represents a promising therapeutic approach for a wide range of diseases that involve exosome‐based therapies.

## 4 Materials and Methods

### 4.1 Cell Culture

Human HEK293T cells (RRID: CVCL_QW54), A549 cells (RRID: CVCL_0023), H1299 cells (RRID: CVCL_0060), HepG2 cells (RRID: CVCL_0027), and BGC‐823 cells (RRID: CVCL_0060) were obtained from National Collection of Authenticated Cell Culture of China and were maintained in DMEM (HyClone, South Logan, UT, USA) containing 10% fetal bovine serum (FBS; Invitrogen, Carlsbad, CA, USA), along with penicillin (100 U/mL) and streptomycin (100 µg/mL) (Invitrogen, Carlsbad, CA, USA). All cells were cultured under standard conditions at 37°C in a humidified incubator with 5% CO_2_.

### 4.2 Generating Brain Metastatic Tumor Cells

Brain metastatic tumor cell lines were generated through serial in vivo selection. Briefly, A549 cells (designated G0 cells) were stably engineered to express firefly luciferase together with an antibiotic resistance gene (Genechem), enabling noninvasive tracking of tumor burden by bioluminescence imaging. Prior to intracardiac inoculation, cells were suspended in PBS at a density of 1–2 × 10^5^ cells per 100 µL. Following anesthesia, 100 µL of the cell suspension was injected into the left ventricle of recipient mice.

Tumor progression was assessed on a weekly basis using a bioluminescence imaging system (Bruker in vivo FX Pro), with D‐luciferin administered intraperitoneally 10 min before image acquisition. Once brain metastatic signals were detected, mice were sacrificed using CO_2_ asphyxiation (28% volume displacement per minute), and brains were aseptically harvested. Metastatic lesions were mechanically dissociated and enzymatically digested in a 1:1 mixture of DMEM and 0.2% collagenase I at 37°C for approximately 1 h, with intermittent gentle mixing. The resulting cell suspension was centrifuged at 1200 rpm for 5 min, followed by brief treatment with 0.25% trypsin for 10 min to obtain single cells. After enzyme neutralization, cells were resuspended in DMEM supplemented with 10% FBS and 1% penicillin–streptomycin and plated in 10 cm^2^ culture dishes. Puromycin selection was applied to enrich for brain metastatic populations, yielding first‐round metastatic cells (G1 cells). These cells were expanded in vitro and subjected to additional rounds of intracardiac injection and recovery to establish G2, G3, and G4 cells.

### 4.3 Plasmid Construction and Cell Transfection

The coding regions of human TEAD1, TEAD2, TEAD3, TEAD4, JUND, MYC, P65, STAT1, STAT3, STAT6, YAP, CTGF, and ANKRD1 were amplified by PCR and subcloned into the pCDH vector (Addgene, #72265) to generate constructs encoding C‐terminal GFP‐tagged fusion proteins. All recombinant plasmids were validated by bidirectional Sanger sequencing. In parallel, gene‐specific shRNAs and a non‐targeting control shRNA were designed, synthesized, and inserted into the pLKO.1 vector according to standard protocols. The primer sequences used for overexpression plasmid construction and the shRNA sequences are provided in Supplementary Table S7.

For stable gene overexpression or knockdown, lentiviral were produced by co‐transfecting the overexpression or shRNA vectors with the packaging plasmids psPAX2 and pMD2.G. Cells were transfected using Lipofectamine 3000 (Invitrogen, Grand Island, NY, USA) in accordance with the manufacturer's protocol. Stable cell populations were subsequently established. The transfection efficiency was confirmed by reverse transcription‐quantitative PCR (RT‐qPCR) and western blot.

### 4.4 Exosome Isolation

Exosomes were isolated from conditioned media collected after 24 h of culture of A549, H1299, HepG2, and BGC‐823 cells maintained in exosome‐depleted, serum‐free medium. The harvested supernatants were sequentially clarified by centrifugation at 1000 × g for 5 min and subsequently at 1000 × g for 30 min at 4°C to eliminate cells and residual debris. The cleared supernatants were then subjected to ultracentrifugation at 100 000 × g for 30 min at 4°C, followed by passage through a 0.22 µm membrane filter. Exosomes were ultimately enriched and collected using a commercial cell culture supernatant exosome isolation kit (Beyotime, #C3620M) according to the manufacturer's instructions.

### 4.5 Exosome Characterization

The morphology and ultrastructural features of isolated exosomes were examined using Cryo‐TEM (Thermo Fisher Scientific, Krios G4). Particle size distribution and concentration were determined by NTA (Particle Metrix GmbH, ZetaView). The presence of canonical exosomal markers (CD63, HSP70, and Alix), together with the absence of Calnexin, was assessed by western blot analysis.

### 4.6 Western Blot Analyses

Total cellular proteins were isolated using RIPA buffer supplemented with a complete protease inhibitor cocktail. Protein lysates were resolved by SDS‐polyacrylamide gel electrophoresis and subsequently transferred onto nitrocellulose membranes. Following membrane blocking to minimize nonspecific binding, the blots were incubated with the indicated primary antibodies. Antibodies against TEAD1 (A13366), JUND (A5433), MYC (A1309), STAT3 (A1193), STAT6 (A19120), phospho‐YAP1‐S127 (AP1398), RAB11 (A20996), CTGF (A11456), and ANKRD1 (A6192) were purchased from ABclonal, while antibodies recognizing TEAD2 (21159‐1‐AP), TEAD3 (13120‐1‐AP), TEAD4 (12418‐1‐AP), actin (66009‐1‐Ig), CD63 (25682‐1‐AP), HSP70 (10995‐1‐AP), Alix (67715‐1‐Ig), Calnexin (10427‐2‐AP), NF‐κB p65 (10745‐1‐AP), STAT1 (66545‐1‐Ig), MST1 (66663‐1‐Ig), MST2 (12097‐1‐AP), LATS1 (17049‐1‐AP), LATS2 (20276‐1‐AP), YAP1 (13584‐1‐AP), TAZ (23306‐1‐AP), SNAP23 (10825‐1‐AP), and CD9 (20597‐1‐AP) were obtained from Proteintech. Horseradish peroxidase‐conjugated secondary antibodies, including goat anti‐mouse IgG H&L (PR30012; Proteintech) and goat anti‐rabbit IgG H&L (PR30011; Proteintech), were used for signal detection.

### 4.7 Chromatin Immunoprecipitation Sequencing and Chromatin Immunoprecipitation‐qPCR

For chromatin immunoprecipitation (ChIP) assays, cells from the indicated experimental groups, including TEAD1‐overexpressing cells and their vector controls, YAP knockdown cells and control cells, as well as YAP knockdown cells subjected to TEAD1 re‐expression together with the appropriate rescue control groups, were subjected to cross‐linking with formaldehyde at a final concentration of 1%. Chromatin was subsequently prepared and immunoprecipitated using a GFP‐tag polyclonal antibody (Proteintech, 50430‐2‐AP), with nonspecific IgG (Santa Cruz Biotechnology, H2615) serving as a negative control. ChIP‐qPCR analysis was carried out using a commercial ChIP assay kit (P2078; Beyotime) following the manufacturer's recommended protocol. Briefly, cross‐linked chromatin was sheared and immunoprecipitated, and the recovered DNA was purified and analyzed by quantitative PCR to assess TEAD1 occupancy at target genomic regions.

### 4.8 ChIP‐seq Analysis

Following quality assessment of the immunoprecipitated DNA, ChIP‐seq libraries were constructed using the NEBNext Ultra II DNA Library Prep Kit (#E7645L; NEB) according to the manufacturer's protocol. Libraries that met quality requirements were pooled and subjected to paired‐end sequencing (PE150) on an Illumina platform at Novogene Bioinformatics Technology Co., Ltd., with sequencing depth determined by effective library concentration and experimental design.

Raw image files generated by the Illumina sequencer were processed through base calling to produce sequencing reads in FASTQ format, which include both nucleotide sequences and corresponding quality scores. Initial quality evaluation of raw reads was performed using Fastp (v0.23.1). Adapter trimming and removal of low‐quality bases were carried out with Trim Galore (v0.6.10). Cleaned reads were subsequently aligned to the human reference genome (hg38) using Bowtie2 (v2.5.1) under default alignment settings. Reads mapping to the mitochondrial genome were excluded from downstream analyses. Alignments with low confidence (MAPQ < 30) and PCR‐derived duplicates were filtered out using SAMtools (v1.17).

Genome‐wide signal tracks in bigWig format were generated using deepTools (v3.5.2) following read normalization. Enriched binding regions were identified by peak calling with MACS2 (v2.2.7.1), applying a false discovery rate threshold of Q < 0.05. Overall data quality was evaluated in accordance with ENCODE ChIP‐seq guidelines, including assessments of library complexity, mapping efficiency, peak characteristics, ENCODE quality metrics, and reproducibility between biological replicates.

### 4.9 Luciferase Assay

To assess the transcriptional regulation of RAB11, CD9, SNAP23, CTGF, and ANKRD1 by TEAD1 and to determine its dependence on YAP, promoter regions of these genes were amplified by PCR using specific primers and inserted into the pGL4‐basic luciferase reporter vector. The empty pGL4‐basic plasmid served as a negative control. H1299 cells stably expressing control vector (OE‐Ctrl) or TEAD1 (OE‐TEAD1), as well as YAP knockdown cells and their corresponding controls, were seeded into 6‐well plates. In parallel, YAP‐depleted cells re‐expressing TEAD1 and matched reconstitution controls were included to evaluate rescue effects. Cells were transfected with the indicated reporter constructs using Lipofectamine 3000 (L3000015; Invitrogen) following the manufacturer's protocol. Firefly luciferase activity was measured 48 h after transfection using a Luciferase Reporter Gene Assay Kit (RG042S; Beyotime). Reporter activity from three independent experiments was quantified and expressed as mean luciferase activity, enabling comparison of TEAD1‐mediated transcriptional activation under YAP‐intact, YAP‐deficient, and TEAD1‐rescued conditions.

### 4.10 Isolation, Culture, and Identification of ADSCs

ADSCs were isolated from healthy C57BL/6 mice. In brief, adipose tissue was collected, rinsed thoroughly with phosphate‐buffered saline (PBS; Meilin, China), and minced into approximately 1 mm^3^ fragments. Tissue fragments were enzymatically digested with 2 mg/mL collagenase type I (Worthington, USA) for 45 min at 37°C. Digestion was terminated by adding an equal volume of DMEM supplemented with 10% fetal bovine serum (FBS) and 1% penicillin–streptomycin. The cell suspension was filtered through a 70 µm mesh and centrifuged at 1500 rpm for 5 min. Red blood cells were lysed using Red Blood Cell Lysis Buffer (Beyotime, China) for 2 min, followed by centrifugation and resuspension in complete medium. Cells were seeded and maintained at 37°C in a humidified incubator. Adherent cells were allowed to expand until reaching 80%–90% confluence, after which they were passaged at a ratio of 1:2 or 1:3. ADSCs were used for downstream experiments after three passages. Cell morphology was monitored using optical microscopy, and the adipogenic differentiation potential was evaluated by Oil Red O staining.

### 4.11 Oil Red O Staining

ADSCs were washed twice with PBS and fixed in 4% paraformaldehyde for 10–20 min at room temperature. Following fixation, the cells were washed again with PBS and incubated with freshly prepared Oil Red O working solution (0.5% Oil Red O in isopropanol, diluted 3:2 with distilled water) for 10–20 min at room temperature. Excess stain was removed by washing the cells with distilled water or 60% isopropanol. Lipid droplet accumulation was visualized and captured using a light microscope.

### 4.12 Isolation, Culture of BMSCs

The BMSCs were cultured from healthy C57BL/6 mice. Femurs and tibiae were harvested, and bone marrow was flushed out using PBS. The collected cells were plated in culture medium containing 10% exosome‐depleted FBS and allowed to adhere at 37°C in a humidified incubator. After 24 h, non‐adherent cells were removed by replacing the medium, and the adherent BMSCs were cultured until reaching 80%–90% confluence. Cells were passaged at a ratio of 1:2 or 1:3 and used for experiments after three passages.

### 4.13 Plasmid Construction and Transfection of ADSCs and BMSCs

ADSCs and BMSCs at passage three were transfected with plasmids encoding TEAD1 overexpression (OE‐TEAD1) or shRNA‐mediated knockdown (sh‐TEAD1), along with the corresponding control vectors. Transfections were performed using Lipofectamine 3000 (Invitrogen) according to the manufacturer's instructions. After selection with puromycin, stable cell lines were established. The efficiency of TEAD1 modulation was verified at both the mRNA level by quantitative real‐time PCR (qPCR) and at the protein level by western blotting.

### 4.14 Co‐Culture of Exosomes and Recipient Cells and Confocal Microscopy Observation

Exosomes were collected from ADSCs or BMSCs stably transfected with TEAD1 overexpression (OE‐TEAD1), shRNA‐mediated knockdown (sh‐TEAD1), or the corresponding control vectors, following the previously described isolation procedure. Recipient cells were seeded onto glass coverslips at a density of 5 × 10^4^ cells per well and allowed to adhere for 12 h at 37°C in a humidified incubator with 5% CO_2_. The medium was then replaced, and exosomes were added to the cultures. Following 24 h of incubation, cells were fixed with 4% paraformaldehyde at room temperature for 10 min and washed three times with PBS. Cell nuclei were stained with DAPI (2‐(4‐aminophenyl)‐1H‐indole‐6‐carboxamidine; D9542‐10MG; Sigma–Aldrich) for 10 min at room temperature, followed by two washes with PBS. Coverslips were mounted on microscope slides and sealed. Immediately, the recipient cell was photographed using a laser confocal scanning microscope (LSM 880, Zeiss, Germany) at 63× magnification.

### 4.15 In Vitro Proliferation Assay

HaCaT cells and HUVECs (1 × 10^3^ cells per well) were seeded into 96‐well plates in a total volume of 100 µL and incubated overnight at 37°C. The following day, cells were treated with exosomes isolated from ADSCs that had been stably transfected with TEAD1 overexpression (OE‐TEAD1), shRNA‐mediated knockdown (sh‐TEAD1), or control vectors. After the designated incubation period with exosomes, 10 µL of CCK‐8 reagent (AC11L054; Life‐iLab, Shanghai, China) was added to each well. Following 1 h of incubation at 37°C, absorbance at 450 nm was measured using a microplate reader (Olympus) to evaluate the proliferation of recipient cells.

### 4.16 Wound‐Healing Assay

HaCaT cells were seeded in six‐well plates at equal densities and cultured until reaching approximately 90% confluence. The cells were then treated with exosomes isolated from ADSCs stably transfected with TEAD1 overexpression (OE‐TEAD1), shRNA‐mediated knockdown (sh‐TEAD1), or the corresponding control vectors (OE‐Ctrl and sh‐Ctrl). Wounds were created using a sterile pipette tip at 0 h. Cells were then rinsed with medium and replaced with fresh medium. The relative area of wound closure was assessed 24 h after the scratch.

### 4.17 Cell Migration Assays

Cell migration was evaluated using 24‐well transwell chambers with 8‐µm pore membranes (Costar, Cambridge, MA, USA). HaCaT cells were pretreated with exosomes derived from ADSCs, and HT22 cells were pretreated with exosomes derived from BMSCs. Both ADSCs and BMSCs were stably transfected with TEAD1 overexpression vectors (OE‐TEAD1), shRNA‐mediated knockdown vectors (sh‐TEAD1), or the corresponding control vectors (OE‐Ctrl and sh‐Ctrl). A total of 1.5 × 10^5^ treated cells were suspended in serum‐free DMEM and added to the upper chamber, while 600 µL of DMEM containing 10% FBS was added to the lower chamber as a chemoattractant. After 48 h of incubation at 37°C, cells that had migrated to the lower surface of the membrane were fixed with 4% paraformaldehyde, stained with crystal violet, and counted under a microscope to assess the effects of exosomes from the four treatment groups on HaCaT and HT22 cell migration.

### 4.18 Tube Formation Assay

The formation of capillary‐like networks by HUVECs was evaluated using a Matrigel‐based tube formation assay (Corning, USA). Cold 96‐well plates were coated with 70 µL of Matrigel per well and evenly distributed on ice. HUVECs were pretreated with exosomes isolated from ADSCs stably transfected with TEAD1 overexpression (OE‐TEAD1), shRNA‐mediated knockdown (sh‐TEAD1), or the corresponding control vectors (OE‐Ctrl and sh‐Ctrl). The treated HUVECs were seeded onto the Matrigel‐coated wells and incubated in culture medium at 37°C for 6 h. Capillary‐like structures were visualized under an optical microscope, and the number of formed capillaries was quantified using ImageJ software (version 1.52a; Media Cybernetics, USA) to assess the effects of exosomes from the four ADSC treatment groups.

### 4.19 Packaging of Adeno‐Associated Virus (AAV) Vectors

AAV‐TEAD1 (serotype 9, U6‐MCS‐CAG‐EGFP, sequence: NM_001166584.2) and AAV‐Ctrl (serotype 9, U6‐MCS‐CAG‐EGFP) used in this study were produced and packaged by Vigene Biosciences (Jinan, China). The titer of AAV‐TEAD1 was determined by quantitative real‐time polymerase chain reaction (qPCR) to be 9.99× 10^13^ v.g./mL.

### 4.20 Mouse Cutaneous Wound Model and Treatment Strategy

#### 4.20.1 Normal Wound Mode

Healthy male BALB/c mice (6 weeks of age, weighing 20–25 g) were used for the in vivo wound‐healing experiments. Mice were anesthetized by intraperitoneal administration of 0.3% phenobarbital sodium (0.1 mL/10 g body weight). After anesthesia, dorsal hair was removed using an electric shaver, followed by depilation with a chemical depilatory cream. A standardized full‐thickness excisional wound with a diameter of 10 mm was then created at the midline of the dorsal skin.

#### 4.20.2 Type 1 Diabetes Mellitus (T1DM) Wound Model

Type 1 diabetes was established in male C57BL/6 mice (6 weeks old, 20–25 g) using a multiple low‐dose STZ protocol. Briefly, mice received intraperitoneal injections of STZ (70 mg/kg; Sigma–Aldrich, Shanghai, China, #S0130) freshly dissolved in citrate buffer, administered once daily for five consecutive days. Control animals received an equivalent volume of vehicle solution. Following a 16‐h fasting period, blood glucose levels were measured from tail vein samples using a handheld glucometer (YuWell, China). Body weight and blood glucose were monitored at baseline and at 5 and 10 days after the final STZ injection. Mice exhibiting FBG concentrations exceeding 16 mmol/L were considered diabetic and included in subsequent experiments. After confirmation of diabetes, a standardized full‐thickness excisional wound (10 mm in diameter) was generated at the dorsal midline of each mouse, following the same surgical procedure described for the non‐diabetic wound model.

#### 4.20.3 Type 2 Diabetes Mellitus (T2DM) Wound Model

Genetic type 2 diabetes was modeled using db/db mice. Mice were 8 weeks old at the onset of the experiments and displayed characteristic metabolic features of T2DM, including obesity (body weight 40–50 g), persistent hyperglycemia (mean blood glucose≈26 mmol/L), and classic diabetic symptoms such as polydipsia, polyuria, and glycosuria. For wound‐healing studies, db/db mice underwent creation of a circular, full‐thickness cutaneous wound with a diameter of 10 mm at the dorsal midline, using the same surgical protocol applied to control and T1DM mice.

#### 4.20.4 Treatment Strategy

For experiments performed in normalwound mode mice, animals were randomly assigned to four treatment groups: (1) TEAD1‐AAV, (2) Ctrl‐AAV, (3) TEAD1‐AAV combined with the exosome secretion inhibitor GW4869 (TEAD1‐AAV+GW4869), and (4) Ctrl‐AAV combined with GW4869 (Ctrl‐AAV+GW4869). Each group included at least five mice. In diabetic wound‐healing models, including both T1DM and T2DM, mice were randomly divided into two groups: (1) TEAD1‐AAV and (2) Ctrl‐AAV, with a minimum of five animals per group.

For all groups, recombinant AAV vectors were administered locally by intradermal injection at the wound margins using a 1‐mL syringe equipped with a 30‐gauge needle. Each wound received four to five evenly distributed injections with a total volume of 20 µL, corresponding to approximately 1 × 10^11^ viral genomes per wound. Wound healing was monitored longitudinally. For normal mice and the T1DM model, wound images were acquired on days 0, 3, 7, 10, and 14 after injury. For the T2DM model, wound closure was documented on days 1, 5, 9, 12, and 14. Digital photographs were analyzed using ImageJ software to calculate wound area, and healing efficiency was expressed as the percentage of wound closure relative to the initial wound size. At day 14 post‐wounding, mice were euthanized, and wound tissues were harvested for subsequent analyses.

### 4.21 Establishment of Contusive Spinal Cord Injury Mouse Model and Experimental Design

A mouse model of spinal cord injury (SCI) was established using 8‐week‐old mice. Animals were anesthetized with isoflurane inhalation, followed by a dorsal midline incision to expose the vertebral column. A laminectomy was performed at the T13 level to fully expose the spinal cord. SCI was induced by applying a complete crush injury to the exposed spinal cord for 2 s using Dumont No. 5 forceps (11295‐00; Fine Science Tools). After injury induction, the paraspinal musculature and skin were sutured in layers. Sham‐operated mice underwent the same surgical exposure and laminectomy procedures without spinal cord compression. Postoperative care included manual bladder expression twice daily until spontaneous or reflexive urination was restored.

Following surgery, mice were randomly assigned to different experimental groups (*n* = 10 per group) according to the study design: (1) TEAD1‐AAV, (2) Ctrl‐AAV, (3) TEAD1‐AAV combined with GW4869 (TEAD1‐AAV+GW4869), and (4) Ctrl‐AAV combined with GW4869 (Ctrl‐AAV+GW4869). TEAD1‐AAV or Ctrl‐AAV was locally administered at the lesion site. For groups receiving exosome inhibition, GW4869 (KM15381; KKL) was administered via intraperitoneal injection at a dose of 2.5 mg/kg. At predetermined time points following SCI, mice were humanely euthanized, and spinal cord segments encompassing the lesion epicenter were collected for subsequent histological, molecular, and functional analyses to evaluate the effects of TEAD1 overexpression and exosome inhibition on spinal cord repair.

### 4.22 Immunohistochemistry (IHC) and Immunofluorescence (IF)

For the wound‐healing model, regenerated skin tissues were collected on day 14 post‐injury. In the SCI model, spinal cord samples were harvested at 4 and 8 weeks following injury.

For IHC, tissue sections were deparaffinized and rehydrated through graded alcohols. Sections were incubated with primary antibodies, followed by treatment with an appropriate secondary antibody and an avidin‐biotin complex (ABC). Staining was visualized using the chromogenic substrate diaminobenzidine (DAB), and images were captured with an optical microscope (Olympus IX70, Tokyo, Japan). Quantitative analysis of IHC signals was performed using ImageJ software.

For IF, deparaffinized and rehydrated sections were blocked with 1.5% goat serum (Merck‐Millipore) and incubated overnight at 4°C with primary antibodies. After washing, sections were incubated with fluorescently labeled secondary antibodies and counterstained with DAPI (D9542‐10MG; Sigma–Aldrich) to visualize nuclei. Fluorescent images were acquired using a fluorescence microscope, and quantification was performed using ImageJ software.

For ex vivo analysis of injured spinal cords, tissues were perfused with 0.9% saline followed by 4% paraformaldehyde, then post‐fixed overnight. Samples were dehydrated, embedded, and sectioned at 10 µm thickness. Sections were blocked with 10% BSA and incubated with primary antibodies overnight at 4°C, followed by secondary antibody incubation at room temperature for 2 h.

Primary antibodies used included TEAD1 (A13366; ABclonal) and IL6 (A21264; ABclonal), as well as CD9 (60232‐1‐Ig; Proteintech), CD90 (66766‐1‐Ig; Proteintech), CD63 (25682‐1‐AP; Proteintech), CD31 (28083‐1‐AP; Proteintech), Ki67 (28074‐1‐AP; Proteintech), and NeuN (66836‐1‐Ig; Proteintech). Fluorescent secondary antibodies were FITC‐conjugated goat anti‐rabbit IgG(H+L) (SA00003‐2; Proteintech) and Cy3‐conjugated goat anti‐mouse IgG(H+L) (SA00009‐1; Proteintech).

### 4.23 Basso Mouse Scale (BMS) Behavioral Analysis

Hind limb motor function was systematically evaluated by measuring multiple parameters, including range of motion, trunk posture, stability, paw placement, toe spacing, tail position, and coordination between forelimbs and hind limbs. Assessments were performed at baseline (pre‐SCI) and at 1, 3, 7, 14, 21, and 28 days post‐SCI. Locomotor performance was quantified using the BMS. Scoring was carried out by one or two trained observers who were blinded to group allocation. Each animal received a score ranging from 0 (complete hind limb paralysis) to 9 (normal locomotion), based on joint movement, paw posture, trunk stability, tail position, and interlimb coordination.

### 4.24 TreadScan Analysis

Gait parameters were assessed using the TreadScan system (CleverSys Inc., Reston, VA), which provides a sensitive, noninvasive method for evaluating limb function under controlled treadmill conditions. Recordings were performed before SCI and at 4 and 8 weeks post‐injury. Each mouse was placed on a transparent treadmill and allowed to walk for 20 s at a speed of 5 cm/s. Movements were captured with a high‐speed digital camera, and the TreadScan software subsequently analyzed individual paw features, including initial contact, stance duration, print length, track width, and toe spread. For each session, four to six consecutive, consistent step cycles were selected for detailed video analysis.

### 4.25 Proteomic Sample Analysis

Cells and exosome samples were lysed in TCEP buffer (2% sodium deoxycholate, 40 mM 2‐chloroacetamide, 100 mM Tris‐HCl, 10 mM tris (2‐chloroethyl) phosphate, 1 mM PMSF, 1 mM protease inhibitor cocktail, pH 8.5) with added phosphatase inhibitors at 99 °C for 30 min. After cooling, samples were digested with trypsin at 37 °C for 16 h. Digestion was quenched with 10% formic acid, centrifuged at 12 000 g for 5 min, and the supernatant was collected and dried in a vacuum concentrator at 45 °C prior to MS analysis.

### 4.26 TF Activity Profiling Sample Preparation

Nuclear extracts were prepared using the NE‐PER kit (#78835; Thermo Scientific) according to the manufacturer's instructions. Biotinylated DNA (3 pmol) was immobilized on Dynabeads M‐280 streptavidin (#14203; Invitrogen) and incubated with nuclear extracts at 4 °C for 2 h. Beads were washed with NETN buffer and PBS to remove unbound proteins, then resuspended in SDS loading buffer and boiled at 95 °C for 5 min. Proteins were separated on 10% SDS‐PAGE gels, stained with Coomassie Brilliant Blue, and subjected to in‐gel trypsin digestion. Peptides were extracted with 0.1% formic acid and 50% acetonitrile, dried, and analyzed by LC‐MS/MS.

### 4.27 Proteome Analysis With Liquid Chromatography‐Tandem Mass Spectrometry

Dried peptides were resuspended in 0.1% formic acid and loaded onto a trap column (100 µm × 2 cm, packed in‐house) to concentrate and desalt the sample. Peptides were then separated on a 150 µm × 30 cm silica microcolumn (particle size 1.9 µm, pore size 120 Å, homemade) using a linear gradient of 4%–99% mobile phase B (80% acetonitrile with 0.1% formic acid) over 150 min at a flow rate of 600 nL/min. Mass spectrometry was performed on an Orbitrap Exploris 480 (Thermo Fisher Scientific) coupled to a Nanoflex nanoelectrospray source, with Field Asymmetric Ion Mobility Spectrometry (FAIMS) applied at –45 V and –65 V to improve selectivity and reduce chemical noise. MS1 scans were acquired at a resolution of 120 000 over a mass range of 350–1600 m/z, with an automatic gain control (AGC) target of 3 × 10^6^ and a maximum injection time of 80 ms. MS2 scans were performed in a data‐dependent manner, with AGC set to 5 × 10^4^ and maximum injection time of 22 ms. Peptides were fragmented using higher‐energy collisional dissociation (HCD), and data acquisition was controlled using Xcalibur software (v4.5).

### 4.28 Peptide Identification and Protein Quantification

Raw MS data were processed using MaxQuant v1.6.17.0 and searched against the human NCBI RefSeq protein database. Carbamidomethylation of cysteine was set as a fixed modification, and oxidation (M) and protein N‐terminal acetylation as variable modifications. A maximum of two missed cleavages was allowed, and identifications were filtered at 1% FDR.

### 4.29 Principal Component Analysis (PCA) and Heatmap

To examine differences in proteomic profiles between groups, PCA was performed using the R package factoextra to visualize sample segregation. Global protein expression data were standardized by converting each value to a z‐score across all samples. Two‐way hierarchical clustering was then applied to the z‐score‐transformed matrix using the pheatmap R package (v1.0.12) to identify patterns of differential protein expression among samples.

### 4.30 Differential Protein and Pathway Enrichment Analysis

Differential expression of proteins between experimental groups was assessed using the Wilcoxon rank‐sum test. *p*‐values were adjusted for multiple comparisons using the Benjamini–Hochberg method to control the false discovery rate. Proteins showing significant differences were further analyzed for functional enrichment. Gene Ontology terms and KEGG pathways were examined using DAVID (https://david.ncifcrf.gov/) or Reactome(https://reactome.org/), with significance defined as *p* < 0.05.

### 4.31 Statistical Analysis

Data collection and analysis were performed using GraphPad Prism 10.0 (GraphPad Software, La Jolla, CA, USA). All quantitative data are presented as the mean ± SD. For comparisons between two groups, Student's two‐tailed *t*‐test was used. For comparisons involving three or more groups, one‐way analysis of variance (ANOVA) was performed, followed by Tukey's post hoc test for multiple comparisons. The precise sample size (*n*) for each experiment is explicitly stated in the corresponding figure legends. A *p*‐value of < 0.05 was considered statistically significant, with significance thresholds set as follows: *p* < 0.05 (∗), *p* < 0.01 (∗∗), *p* < 0.001 (∗∗∗), *p* < 0.0001 (∗∗∗∗).

## Author Contributions

W.Y. and C.D. contributed to conceptualization; Y.P. and Y.W. performed all the cellular and animal experiments with the help of B.L., H.Z., L.L., and J.W; Y.P., Y.W., and W.S. contributed to data quality control and analysed the data; W.G., C.D., and W.Y. supervised the study; C.D., Y.P., Y.W., and W.S. wrote the manuscript. All the authors have read and approved the final manuscript.

## Ethics Statement

All animal protocols were approved by the Institutional Animal Care and Use Committee of Fudan University (Ethical approval ID IDM2024043a).

## Conflicts of Interest

The authors declare no conflicts of interest.

## Supporting information




**Supporting File 1**: advs74490‐sup‐0001‐SuppMat.docx.

**Supporting File 2**: advs74490‐sup‐0002‐TableS1.xlsx.

**Supporting File 3**: advs74490‐sup‐0003‐TableS2.xlsx.

**Supporting File 4**: advs74490‐sup‐0004‐TableS3.xlsx.

**Supporting File 5**: advs74490‐sup‐0005‐TableS4.xlsx.

**Supporting File 6**: advs74490‐sup‐0006‐TableS5.xlsx.

**Supporting File 7**: advs74490‐sup‐0007‐TableS6.xlsx.

**Supporting File 8**: advs74490‐sup‐0008‐TableS7.xlsx.

## Data Availability

The data that support the findings of this study are available in the supplementary material of this article.
