# Supplementary material for: TEAD1 Enhances Exosome Secretion and Promotes Exosome‐Mediated Tissue Regeneration
Source: Adv Sci (Weinh). 2026 Mar 12;13(25):e14104. doi: 10.1002/advs.202514104 (PMC13137834; doi:10.1002/advs.202514104)
Supplement: Supplementary file 1 — Supporting File 1: advs74490‐sup‐0001‐SuppMat.docx. [file ADVS-13-e14104-s008.docx]

**TEAD1 Enhances Exosome Secretion and Promotes Exosome-Mediated Tissue Regeneration**

Yan Pu^1,#^, Yi Wan^1,#^, Wenhao Shi^2,#^, Bin Li^3^, Haiyue Zhang^4^, Jun Wu^5^, Lingling Li^1^, Wenjia Guo^4,*^, Chen Ding^1,4*^ and Wenjun Yang^6,*^


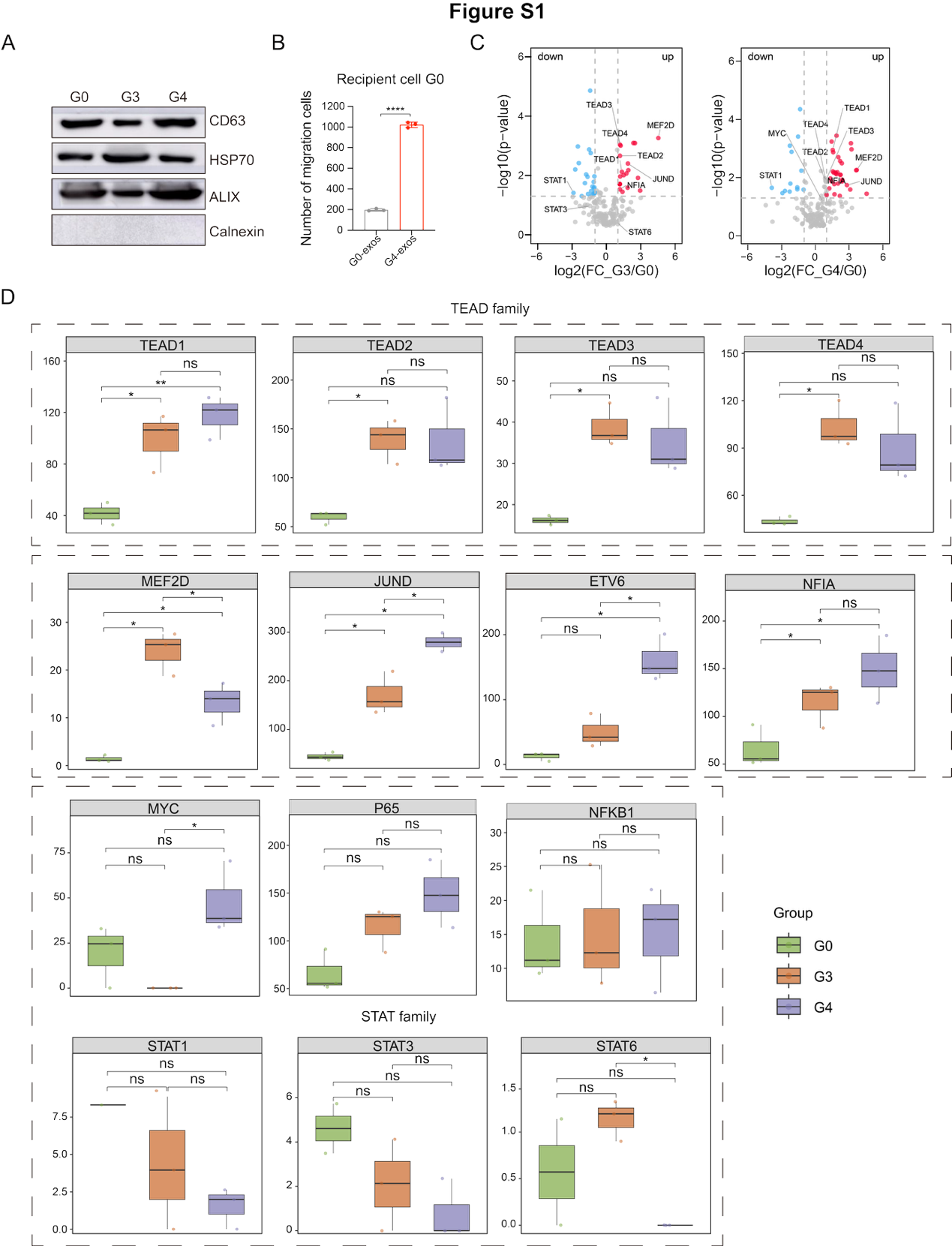


**Figure S1** Supplementary analysis of protein abundance changes and exosome-related effects upon TEAD1 over-expression, related to Figure 1. **A.** Western blotting analysis of exosome-specific markers CD63, HSP70, ALIX, and Calnexin. **B.** Transwell assays assessing cell migration. The exosomes derived from the G4 cell line demonstrated significantly higher migration capacity than exosomes derived from the G0 cell line (n = 3 per group). **C.** Comparison of transcription factor activities among lung cancer cell lines of different malignancies (G0, G3, and G4). The figure shows the comparison of transcription factor activities between G3 and G4 cells versus G0 cells. **D.** Boxplots show transcription factor activities across different generations (G0, G3, and G4; n = 3 per group, mean ± SD). Differences among the groups were determined with one‐way ANOVA with Tukey's posttest and Student's two-tailed t-test. Data were considered statistically significant when *P* < 0.05 (∗), *P* < 0.01 (∗∗), and *P* < 0.0001 (∗∗∗∗) versus the indicated group.


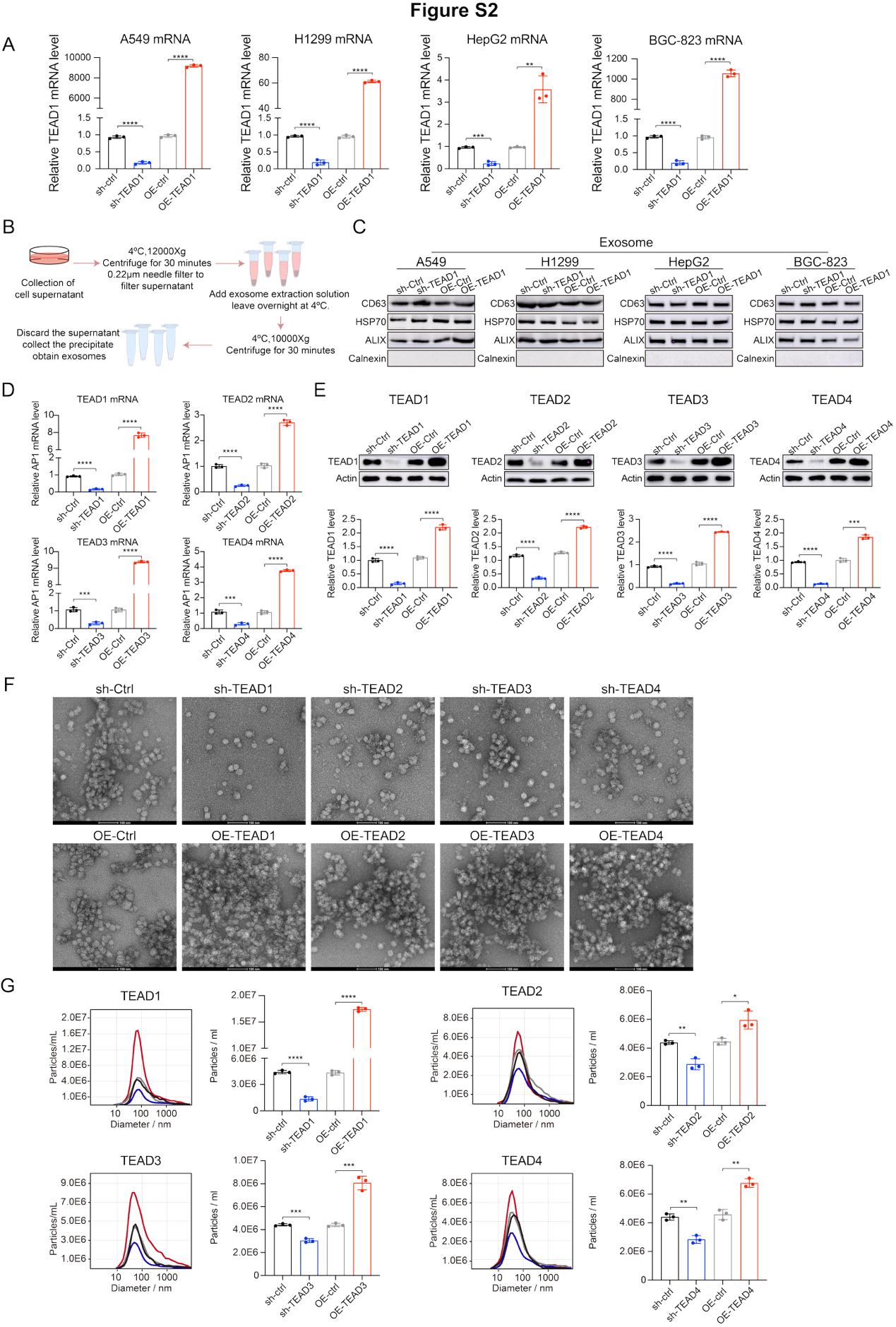


**Figure S2** Supplementary analysis of protein abundance changes and exosome-related effects upon TEAD1 over-expression, related to Figure 1. **A.** Q-PCR validation of TEAD1 overexpression and Knockdown in four different tumor cell lines (n = 3 per group, mean ± SD). **B.** Schematic diagram of the exosome extraction process. **C.** Western blot analysis of exosome-specific markers CD63, HSP70, ALIX, and Calnexin, after TEAD1 knockdown and overexpression in four different cell lines. **D-E.** Q-PCR (D) and Western blot (E) analyses validating the overexpression and knockdown of TEAD1, TEAD2, TEAD3, and TEAD4 in A549 cells (n = 3 per group, mean ± SD). **F.** TEM images of exosomes after TEAD1, TEAD2, TEAD3, and TEAD4 knockdown and overexpression in A549 cells. Scale bar = 100 nm. **G.** Statistics of exosome size and particle number after TEAD1, TEAD2, TEAD3, and TEAD4 knockdown and overexpression in A549 cells (n = 3 per group, mean ± SD). Differences among the groups were determined with one‐way ANOVA with Tukey's posttest and Student's two-tailed t-test. Data were considered statistically significant when *P* < 0.05 (∗), *P* < 0.01 (∗∗), *P* < 0.001 (∗∗∗), and *P* < 0.0001 (∗∗∗∗) versus the indicated group.


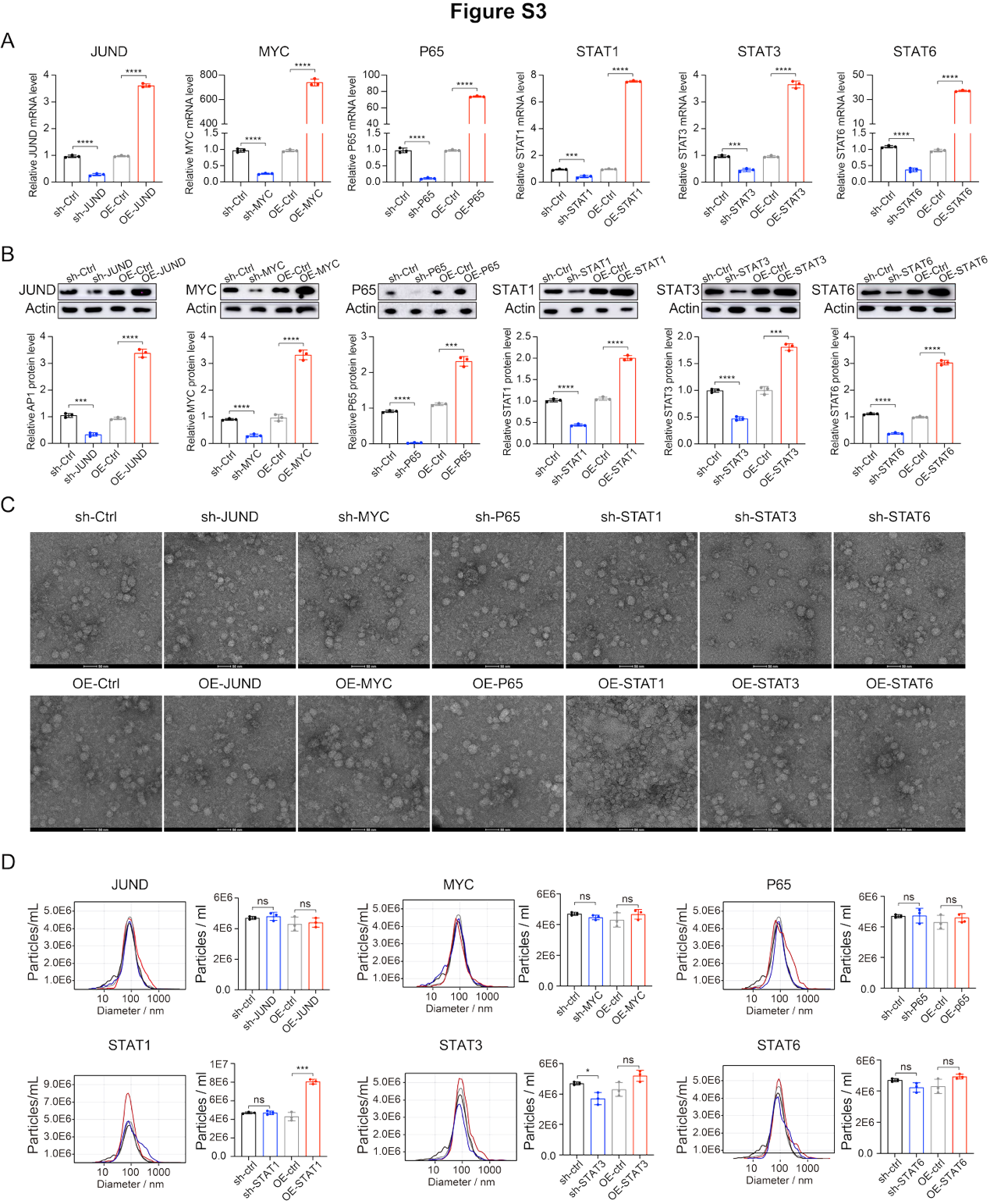


**Figure S3** Supplementary analysis of functional validation of other transcription factors in exosome secretion, related to Figure 1. **A-B.** Q-PCR (A) and Western blot (B) analyses validating the overexpression and knockdown of six transcription factors in A549 cells (n = 3 per group, mean ± SD). **C.** TEM images of exosomes after six transcription factors knockdown and overexpression in A549 cells. Scale bar = 50 nm. **D.** Statistics of exosome size and particle number after six transcription factors knockdown and overexpression in A549 cells (n = 3 per group, mean ± SD). Differences among the groups were determined with one‐way ANOVA with Tukey's posttest and Student's two-tailed t-test. Data were considered statistically significant when *P* < 0.05 (∗), *P* < 0.01 (∗∗), *P* < 0.001 (∗∗∗), and *P* < 0.0001 (∗∗∗∗) versus the indicated group.

**
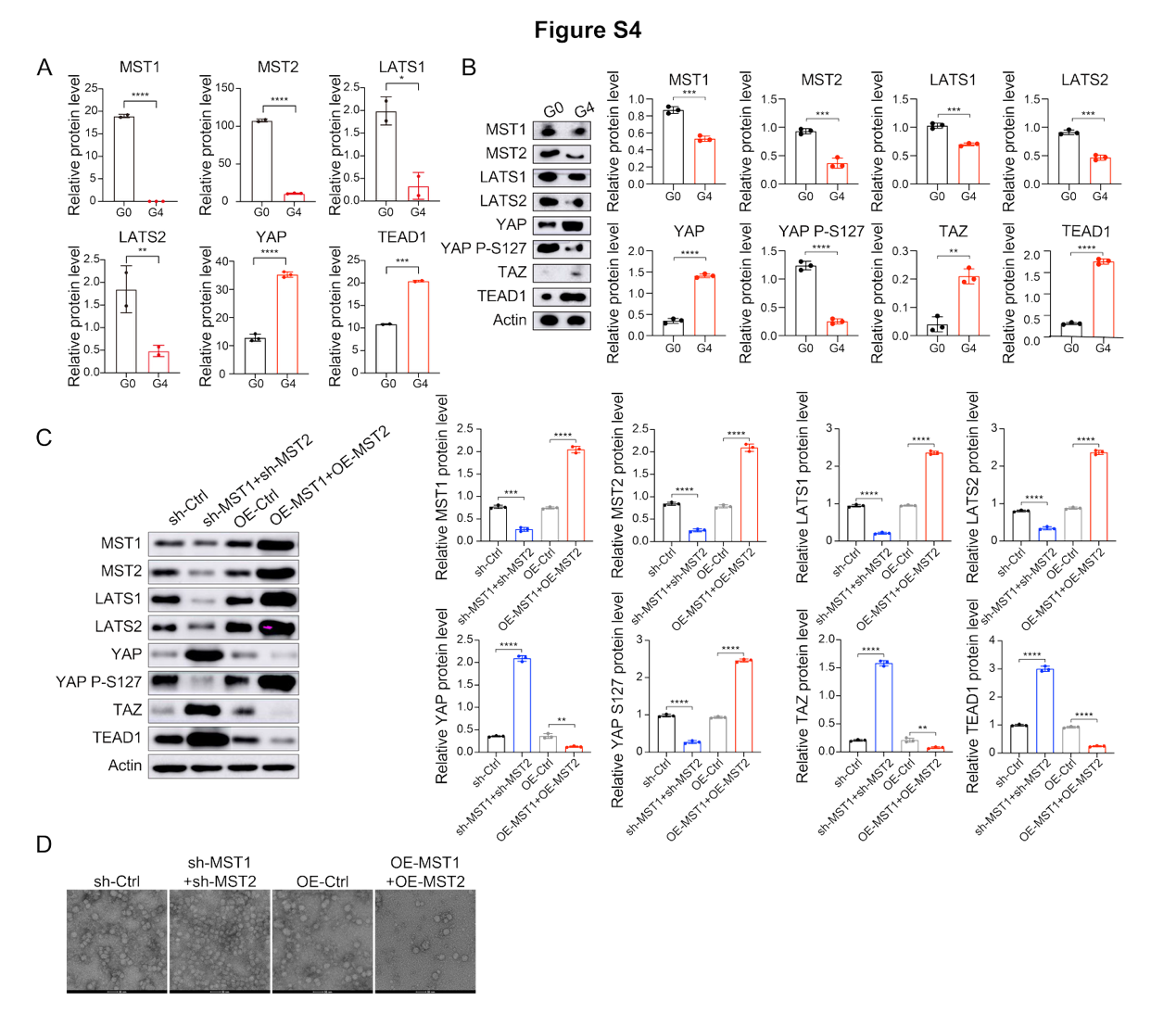
**

**Figure S4** Supplementary analysis of protein levels of upstream regulators of the Hippo pathway, related to Figure 1. **A.** The protein levels of upstream regulators and downstream effectors of the Hippo pathway in G0 and G4 cells (n = 3 per group, mean ± SD). **B.** Western blot validation of upstream regulators and downstream effectors of the Hippo signaling pathway in G0 and G4 cells (n = 3 per group, mean ± SD). **C.** Western blot analysis of Hippo components and TEAD1 in H1299 cell lines after MST1/2 knockdown or overexpression (n = 3 per group, mean ± SD). **D.** TEM images of exosomes in H1299 cell lines after MST1/2 knockdown or overexpression. Scale bar = 50 nm. Differences among the groups were determined with one‐way ANOVA with Tukey's posttest and Student's two-tailed t-test. Data were considered statistically significant when *P* < 0.05 (∗), *P* < 0.01 (∗∗), *P* < 0.001 (∗∗∗), and *P* < 0.0001 (∗∗∗∗) versus the indicated group.


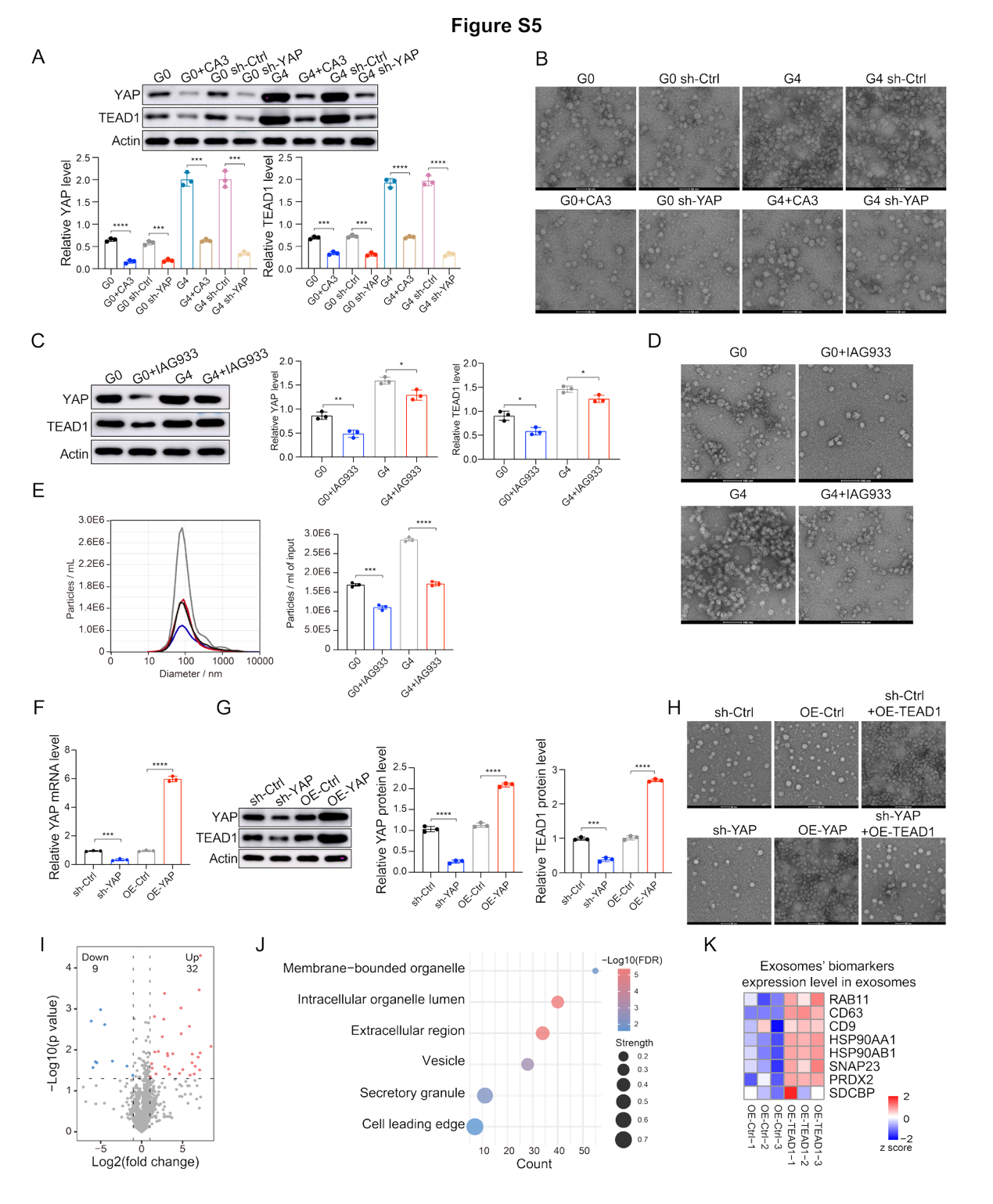


**Figure S5** Supplementary analysis of YAP/TAZ inhibition or YAP knockdown reduces exosome secretion in G0 and G4 lung cancer cells, related to Figure 1.**A.** Western blot analysis of YAP and TEAD1 in G0 and G4 cell lines after CA3 treatment or YAP knockdown (n = 3 per group, mean ± SD). **B.** TEM images of exosomes in G0 and G4 cell lines after CA3 treatment or YAP knockdown, Scale bar = 50 nm. **C.** Western blot analysis of YAP and TEAD1 in G0 and G4 cell lines after IAG933 treatment (n = 3 per group, mean ± SD). **D.** TEM images of exosomes in G0 and G4 cell lines after IAG933 treatment. Scale bar = 100 nm. **E.** Statistics of exosome size and particle number in G0 and G4 cell lines after IAG933 treatment (n = 3 per group, mean ± SD). **F-G.** Quantitative real-time reverse transcription PCR (qRT-PCR) (F) and Western blot analysis (G) of YAP in H1299 cell lines after YAP knockdown and overexpression (n = 3 per group, mean ± SD). **H.** TEM images of exosomes after TEAD1 overexpression in sh-YAP or OE-YAP H1299 cell lines. Scale bar = 50 nm. **I.** Differential protein expression in exosomes isolated from OE-TEAD1 and OE-Ctrl groups. Red indicates up-regulated proteins, while blue indicates down-regulated ones. **J.** Up-regulated signaling pathways in exosomes isolated from OE-TEAD1 and OE-Ctrl groups. **K.** The heatmap showed exosomes’ biomarkers expression level in exosomes (n = 3 per group). Differences among the groups were determined with one‐way ANOVA with Tukey's posttest and Student's two-tailed t-test. Data were considered statistically significant when *P* < 0.05 (∗), *P* < 0.01 (∗∗), *P* < 0.001 (∗∗∗), and *P* < 0.0001 (∗∗∗∗) versus the indicated group.


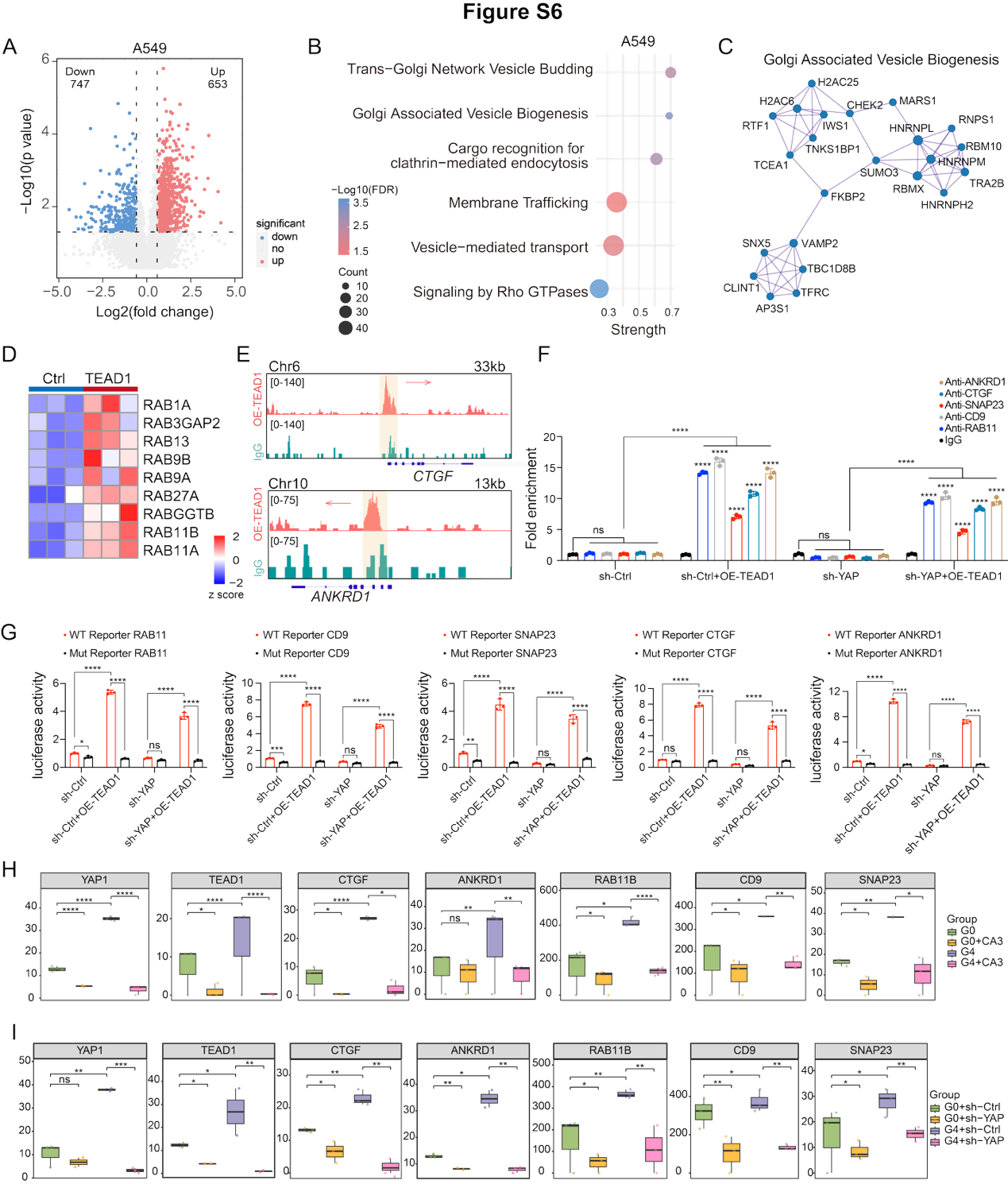


**Figure S6** Supplementary analysis of Proteomic analysis of changes in lung cancer cell lines and exosome-related effects upon TEAD1 overexpression, related to Figure 1. **A.** Protein abundance changes in A549 cell lines upon TEAD1 overexpression. Red indicates up-regulated transcription factors, while blue indicates down-regulated ones. **B.** Up-regulated signaling pathways in A549 cells overexpressing TEAD1. **C.** Protein-protein interaction network associated with Golgi-associated vesicle Biogenesis. **D.** Abundance distribution of RAB family proteins regulating exosome secretion (n = 3 per group). **E.** ChIP sequencing illustrating the binding of TEAD1 to the promoter of CTGF, and ANKRD1 (n = 3 per group, mean ± SD). **F.** Chromatin immunosuppression (ChIP)-quantitative polymerase chain reaction illustrating the binding of TEAD1 to the promoter of RAB11, CD9, SNAP23, CTGF, and ANKRD1 (n = 3 per group, mean ± SD). **G.** Luciferase reporter assay of sh-YAP H1299 cells transfected with RAB11, CD9, SNAP23, CTGF, and ANKRD1 overexpression vector or empty vector control (n = 3 per group, mean ± SD). **H.** CA3 treatment significantly reduced YAP, TEAD1, and its downstream target genes in both G0 and G4 cells (n = 3 per group, mean ± SD). **I.** YAP knockdown significantly reduced TEAD1 and its downstream target genes in both G0 and G4 cells (n = 3 per group, mean ± SD). Differences among the groups were determined with one‐way ANOVA with Tukey's posttest and Student's two-tailed t-test. Data were considered statistically significant when *P* < 0.05 (∗), *P* < 0.01 (∗∗), *P* < 0.001 (∗∗∗), and *P* < 0.0001 (∗∗∗∗) versus the indicated group.


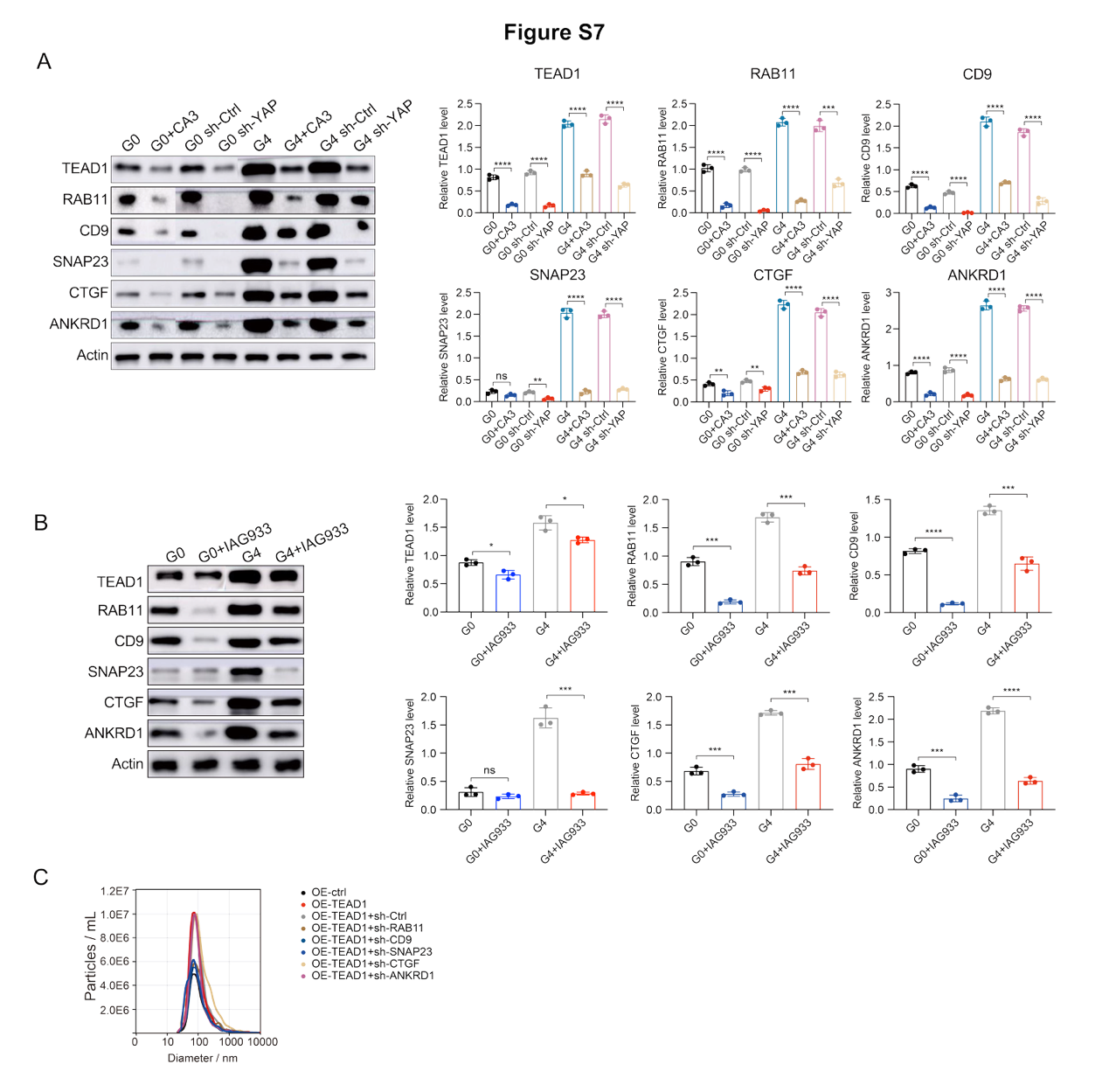


**Figure S7** Supplementary analysis of YAP/TAZ inhibition or YAP knockdown downregulates TEAD1 and its downstream target genes in G0 and G4 cells, related to Figure 2. **A.** Western blot analysis of TEAD1 and its downstream effectors in G0 and G4 cell lines after CA3 treatment or YAP knockdown (n = 3 per group, mean ± SD). **B.** Western blot analysis of TEAD1 and its downstream effectors in G0 and G4 cell lines after IAG933 treatment (n = 3 per group, mean ± SD). **C.** Statistics of exosome size and particle number after RAB11, CD9, SNAP23, CTGF, and ANKRD1 knockdown in OE-TEAD1 H1299 cell lines. Differences among the groups were determined with one‐way ANOVA with Tukey's posttest and Student's two-tailed t-test. Data were considered statistically significant when *P* < 0.05 (∗), *P* < 0.01 (∗∗), *P* < 0.001 (∗∗∗), and *P* < 0.0001 (∗∗∗∗) versus the indicated group.


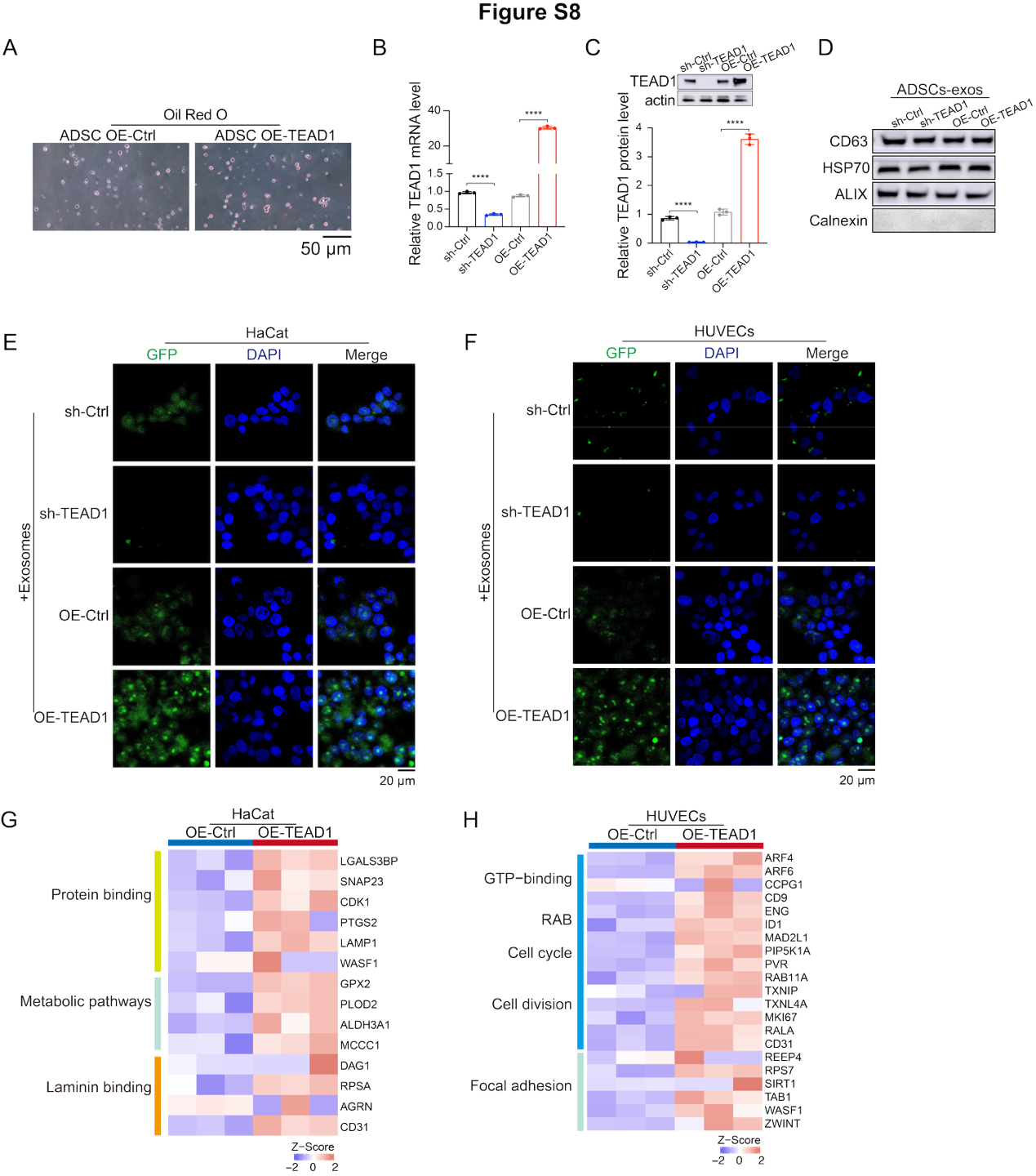


**Figure S8** Supplementary analysis of TEAD1-mediated enhancement of exosome secretion promotes proliferation of HaCaT cells and angiogenesis of HUVECs, related to Figure 3. **A.** Oil red O staining demonstrates ADSCs isolated from primary mice under bright field images, scale bar = 50 μm. **B.** Q-PCR validation of TEAD1 overexpression and knockdown in ADSCs (n = 3 per group, mean ± SD). **C.** Western blot validation of TEAD1 overexpression and knockdown in ADSCs (n = 3 per group, mean ± SD). **D.** Western blotting analysis of exosome-specific markers CD63, HSP70, ALIX, and Calnexin in ADSCs-exos. **E.** Fluorescence microscopy showed the localization of TEAD1 in HaCat cells after treatment with OE-TEAD1 ADSCs-exos or sh-TEAD1 ADSCs-exos, scale bar = 20 μm. **F.** Fluorescence microscopy showed the localization of TEAD1 in HUVECs after treatment with OE-TEAD1 ADSCs-exos or sh-TEAD1 ADSCs-exos, scale bar = 20 μm. **G.** The heatmap showing the differentially expressed proteins in HaCat cells treated with OE-TEAD1 ADSCs-exos or OE-Ctrl ADSCs-exos, the expression of proteins participating in protein binding, metabolic pathways, and laminin binding in HaCat cells is shown (n = 3 per group). **H.** The heatmap showing the differentially expressed proteins in HUVECs treated with OE-TEAD1 ADSCs-exos or OE-Ctrl ADSCs-exos, the expression of proteins participating in GTP-binding, RAB, cell cycle, cell division, and focal adhesion in HUVECs is shown (n = 3 per group). Differences among the groups were determined with one‐way ANOVA with Tukey's posttest and Student's two-tailed t-test. Data were considered statistically significant when *P* < 0.0001 (∗∗∗∗) versus the indicated group.

**
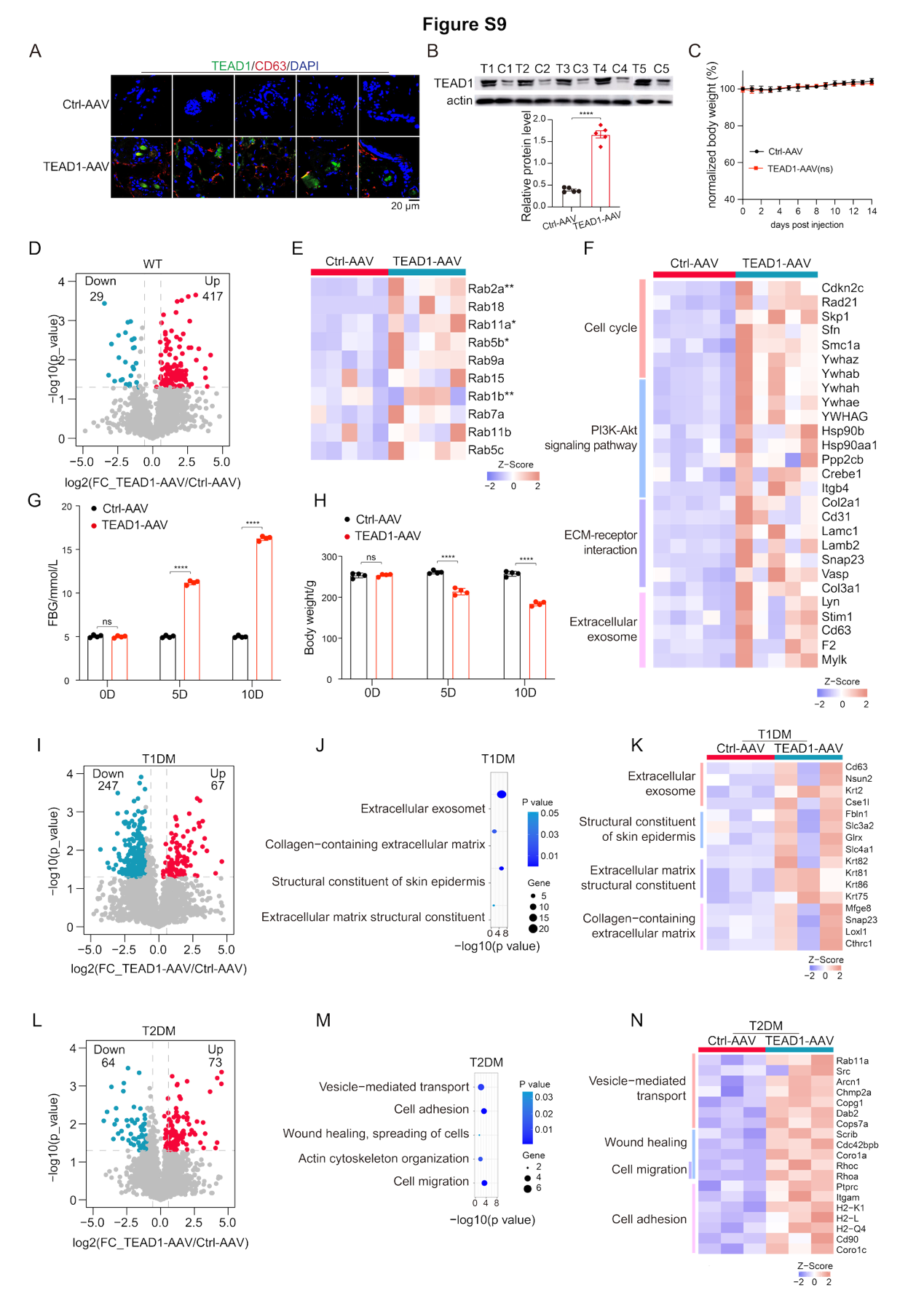
**

**Figure S9** Supplementary analysis of TEAD1-AAV gene therapy enhances skin wound healing, related to Figure 4. **A.** Immunofluorescence staining (IF) was performed to assess the expression of TEAD1 and CD63 in T1DM mice treated with TEAD1-AAV or Ctrl-AAV at day 14, scale bar, 20 µm. **B.** Western blotting analysis confirmed successful overexpression of TEAD1 in the wound tissue of the TEAD1-AAV group (n = 5 per group, mean ± SD). **C.** Measurements of body weight (measured each day and normalized to the averaged weight of day 0; n = 5 per group, mean ± SD). **D.** Differential protein expression in the TEAD1-AAV group. Red indicates up-regulated proteins, while blue indicates down-regulated ones. **E.** The heatmap showing the differentially expressed proteins in the TEAD1-AAV group and the Ctrl-AAV group associated with the RAB family (n = 5 per group). **F.** The heatmap showing the differentially expressed proteins in the TEAD1-AAV group and the Ctrl-AAV group, annotated for representative pathways (n = 5 per group). **G.** The FBG of the control group and STZ group was detected via blood glucose test strips in mice on the 0, 5^th^, and 10^th^ day. The mice without STZ injection were utilized as controls (n = 4 per group, mean ± SD). **H.** The body weight of the Control group and the STZ group was measured by an electronic weighing scale in mice on the 0, 5^th^, and 10^th^ day (n = 4 per group, mean ± SD). **I.** Volcano plot showing the protein expression between the TEAD1-AAV and the Ctrl-AAV groups in TIDM mice. **J.** Up-regulated signaling pathways in the TEAD1-AAV group in TIDM mice. **K.** The heatmap showing the differentially expressed proteins in T1DM mice treated with TEAD1-AAV or Ctrl-AAV, the expression of proteins participating in extracellular exosome, structural constituent of skin epidermis, extracellular matrix structural constituent, and collagen-containing extracellular matrix in T1DM mice is shown (n = 3 per group). **L.** Volcano plot showing the protein expression between the TEAD1-AAV and the Ctrl-AAV groups in T2DM mice. **M.** Up-regulated signaling pathways in the TEAD1-AAV group in T2DM mice. **N.** The heatmap showing the differentially expressed proteins in T2DM mice treated with TEAD1-AAV or Ctrl-AAV, the expression of proteins participating in vesicle-mediated transport, wound healing, cell migration, and cell adhesion in T2DM mice is shown (n = 3 per group). Differences among the groups were determined with one‐way ANOVA with Tukey's posttest and Student's two-tailed t-test. Data were considered statistically significant when *P* < 0.05 (∗), *P* < 0.01 (∗∗), and *P* < 0.0001 (∗∗∗∗) versus the indicated group.


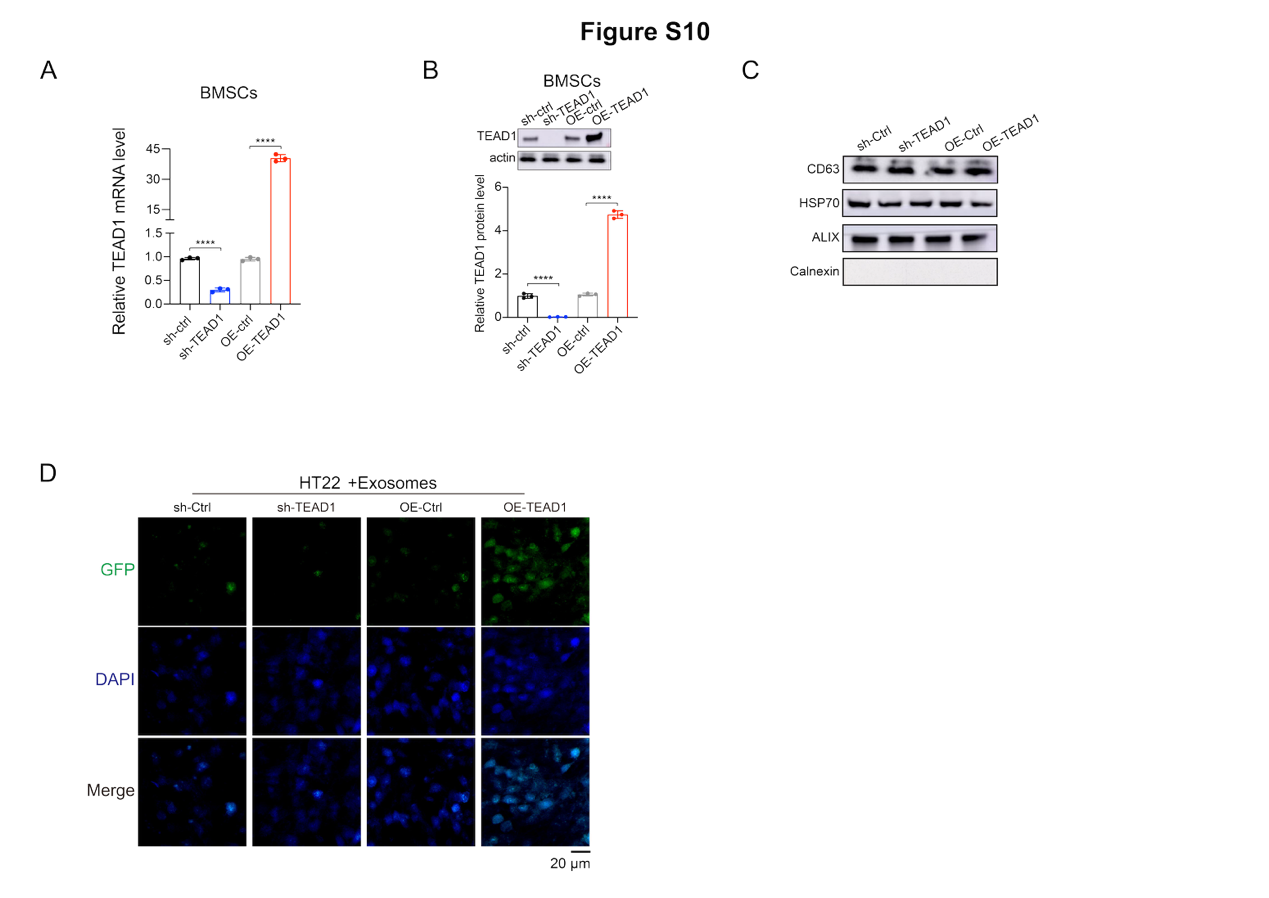


**Figure S10** Supplementary analysis of TEAD1 enhances BMSC-Exos secretion and promotes neural repair, related to Figure 6. **A.** Q-PCR validation of TEAD1 overexpression and knockdown in BMSCs (n = 3 per group, mean ± SD). **B.** Western blot validation of TEAD1 overexpression and knockdown in BMSCs (n = 3 per group, mean ± SD). **C.** Western blotting analysis of exosome-specific markers CD63, HSP70, ALIX, and Calnexin in BMSCs-exos. **D.** Fluorescence microscopy showed the localization of TEAD1 in HT22 cells after treatment with OE-TEAD1 BMSCs-exos or sh-TEAD1 BMSCs-exos, scale bar = 20 μm. Differences among the groups were determined with one‐way ANOVA with Tukey's posttest and Student's two-tailed t-test. Data were considered statistically significant when *P* < 0.0001 (∗∗∗∗) versus the indicated group.


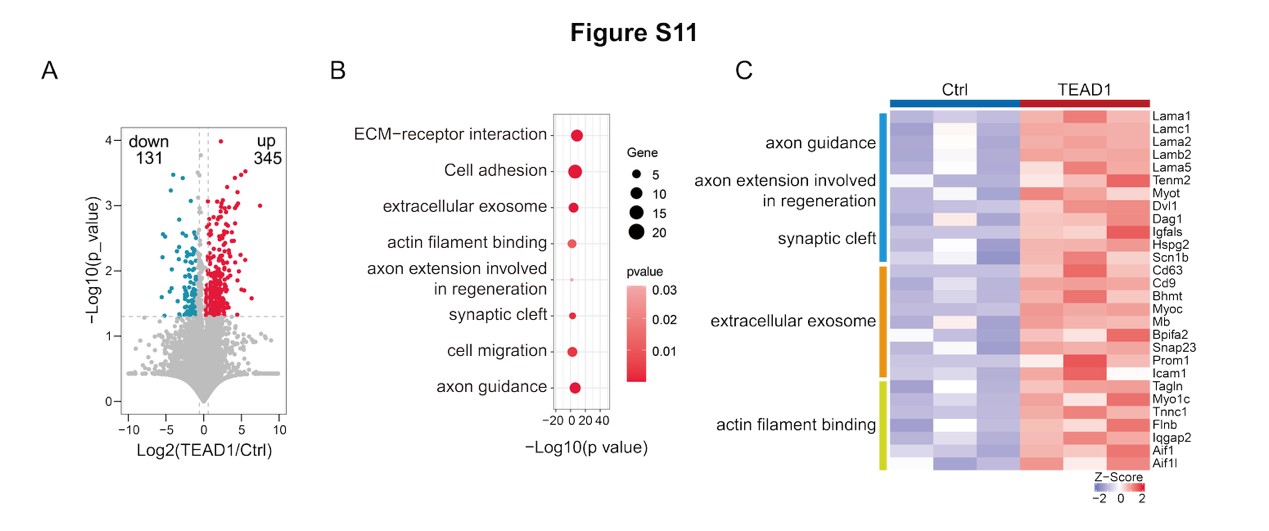


**Figure S11** Supplementary analysis of TEAD1-AAV therapy enhances exosome secretion and facilitates functional recovery after SCI, related to Figure 7. **A.** Volcano plot showing the protein expression between the TEAD1-AAV and the Ctrl-AAV groups. **B.** Up-regulated signaling pathways in the TEAD1-AAV group. **C.** The heatmap showing the differentially expressed proteins in the TEAD1-AAV group and the Ctrl-AAV group, annotated for representative pathways (n = 3 per group). Differences among the groups were determined with Wilcoxon rank-sum test.

**
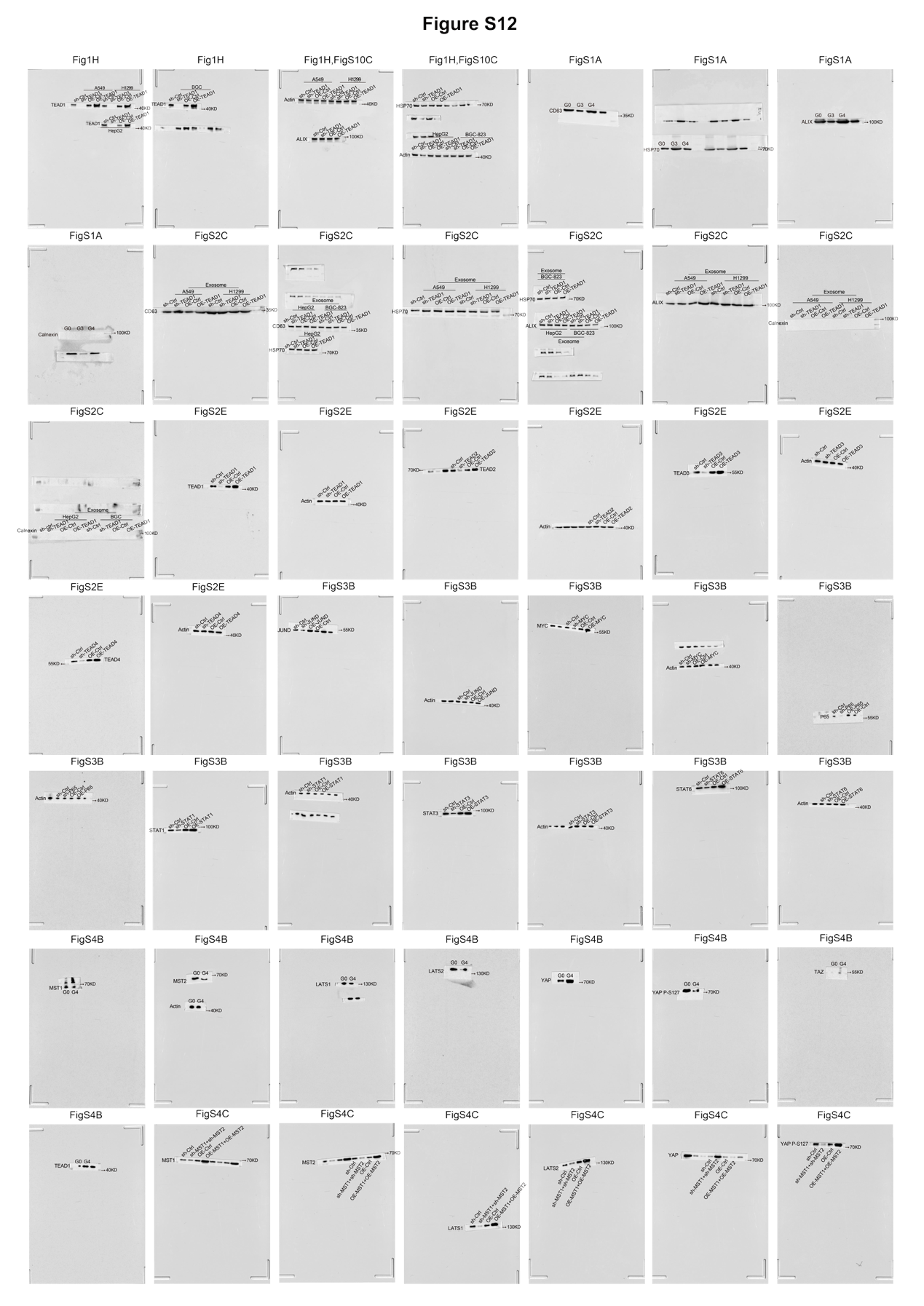
**

**
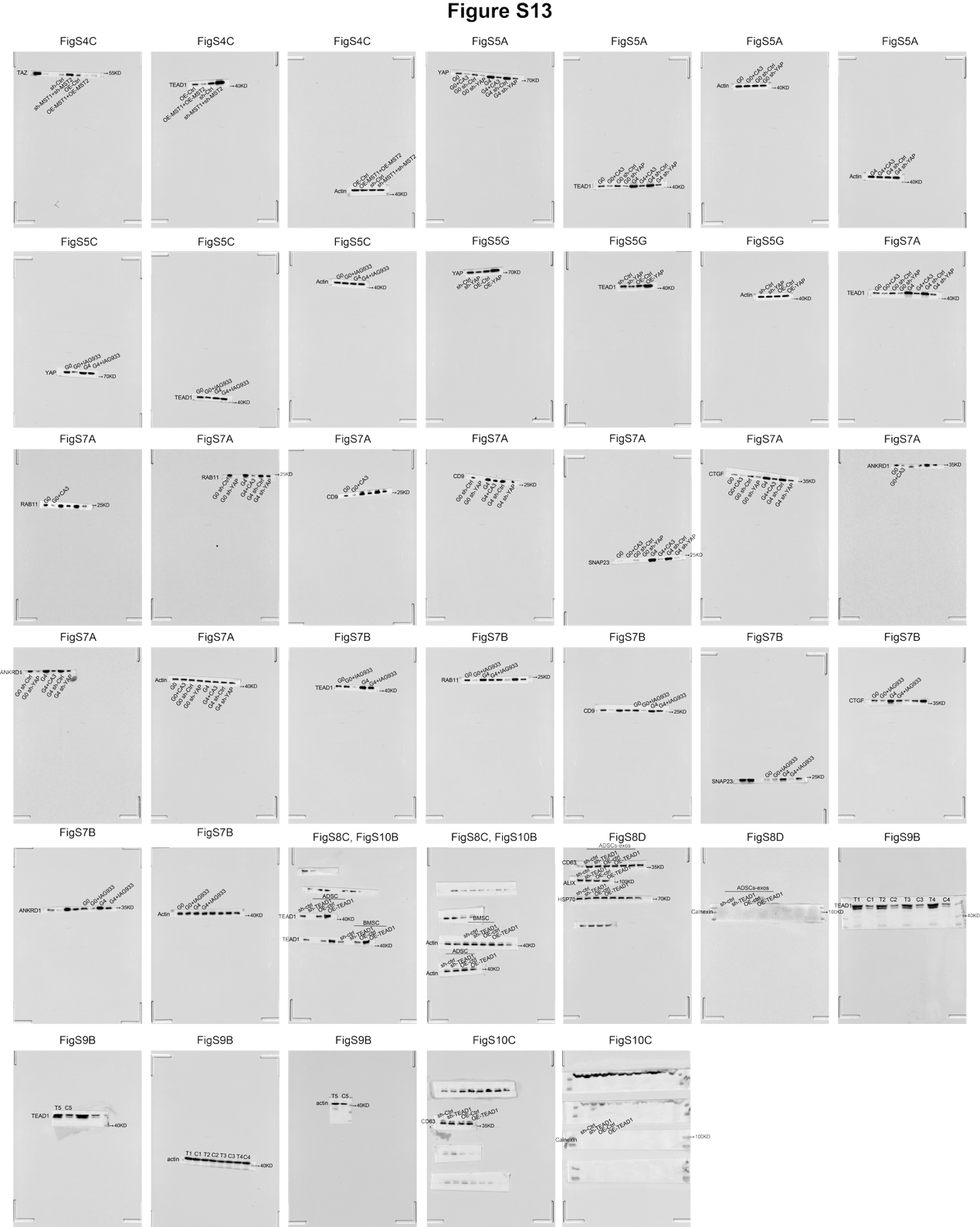
**

**Figure S12-13** The original raw western blotting data.
